# Molecular insights into how a deficiency of amylose affects carbon allocation – carbohydrate and oil analyses and gene expression profiling in the seeds of a rice waxy mutant

**DOI:** 10.1186/1471-2229-12-230

**Published:** 2012-12-05

**Authors:** Ming-Zhou Zhang, Jie-Hong Fang, Xia Yan, Jun Liu, Jin-Song Bao, Gunnel Fransson, Roger Andersson, Christer Jansson, Per Åman, Chuanxin Sun

**Affiliations:** 1 College of Life Science, China JiLiang University, Hangzhou, 310018, China; 2 Department of Plant Biology & Forest Genetics, Uppsala BioCenter, Swedish University of Agricultural Sciences and Linnean Center for Plant Biology, P.O. Box 7080, SE, 75007, Uppsala, Sweden; 3 Heihe Key Laboratory of Ecohydrology and Integrated River Basin Science, Cold and Arid Regions Environmental and Engineering Institute, Chinese Academy of Sciences, 260 Donggang West Road, Lanzhou, 730000, China; 4 Institute of Nuclear Agricultural Sciences, Zhejiang University, Hangzhou, Zhejiang, 310029, China; 5 Department of Food Science, Uppsala BioCenter, Swedish University of Agricultural Sciences, P.O. Box 7051, SE, 75007, Uppsala, Sweden; 6 Lawrence Berkeley National Laboratory, Earth Sciences Division, 1 Cyclotron Road, Berkeley, CA, 94720, U.S.A

**Keywords:** Carbon allocation, Ric*e (Oryza sativa*), Waxy seeds, Suppression subtractive hybridization (SSH), Quantitative polymerase chain reaction (qPCR), Gene expression

## Abstract

**Background:**

Understanding carbon partitioning in cereal seeds is of critical importance to develop cereal crops with enhanced starch yields for food security and for producing specified end-products high in amylose, β-glucan, or fructan, such as functional foods or oils for biofuel applications. Waxy mutants of cereals have a high content of amylopectin and have been well characterized. However, the allocation of carbon to other components, such as β-glucan and oils, and the regulation of the altered carbon distribution to amylopectin in a waxy mutant are poorly understood. In this study, we used a rice mutant, *GM077*, with a low content of amylose to gain molecular insight into how a deficiency of amylose affects carbon allocation to other end products and to amylopectin. We used carbohydrate analysis, subtractive cDNA libraries, and qPCR to identify candidate genes potentially responsible for the changes in carbon allocation in *GM077* seeds.

**Results:**

Carbohydrate analysis indicated that the content of amylose in *GM077* seeds was significantly reduced, while that of amylopectin significantly rose as compared to the wild type BP034. The content of glucose, sucrose, total starch, cell-wall polysaccharides and oil were only slightly affected in the mutant as compared to the wild type. Suppression subtractive hybridization (SSH) experiments generated 116 unigenes in the mutant on the wild-type background. Among the 116 unigenes, three, *AGP*, *ISA1* and *SUSIBA2-like,* were found to be directly involved in amylopectin synthesis, indicating their possible roles in redirecting carbon flux from amylose to amylopectin. A bioinformatics analysis of the putative SUSIBA2-like binding elements in the promoter regions of the upregulated genes indicated that the SUSIBA2-like transcription factor may be instrumental in promoting the carbon reallocation from amylose to amylopectin.

**Conclusion:**

Analyses of carbohydrate and oil fractions and gene expression profiling on a global scale in the rice waxy mutant *GM077* revealed several candidate genes implicated in the carbon reallocation response to an amylose deficiency, including genes encoding AGPase and SUSIBA2-like. We believe that *AGP* and *SUSIBA2* are two promising targets for classical breeding and/or transgenic plant improvement to control the carbon flux between starch and other components in cereal seeds.

## Background

Cereal crops are of critical importance in agriculture. The top three cereals in global production (2009) are maize, wheat, and rice, with 819, 686 and 685 M tonnes, respectively ( http://faostat.fao.org). Cereal crops constitute our largest primary food source and are also highly used in food and non-food industrial applications. Contributing factors to the importance of cereals are that they can be bred to be very high yielding, that cereal grains lend themselves to long-term storage, and that the grain can accumulate different types of carbohydrates and lipids. Major carbohydrates in cereal caryopses are the starch components amylose and amylopectin, cell wall components, such as different types of arabinoxylan, mixed-linkage β-glucan and cellulose, fructooligosaccharides, fructan, and sucrose [1,2]. Interestingly significant amounts of oil can also be stored in the endosperm, especially in oats [3]. The composition of the cereal grain dictates the end use of the crop. For example, the cereal endosperm is the most important source of starch worldwide [4,5] and is therefore of tremendous value for food security. There is an ongoing search for genotypes with high content of amylose, β-glucan and/or fructan for different applications within the functional food sector [6-8]. At the other end of the spectrum are efforts to develop cereals that redirect carbon flux from carbohydrates to oils for production of high-density biofuels [9-13]. A thorough understanding of the mechanisms for the partitioning of photosynthates in cereals is crucial for our ability to boost starch yield, to develop specialty crops for the functional food industry, such as barley with enhanced ß-glucan levels, and to tailor cereal production for the non-food industry.

Carbon partitioning in higher plants has been studied at the whole-plant level [14,15], for certain types of plant tissues [16-18], and for plant cells [19]. However, many questions remain unanswered. For example, we need to identify and map the actions of key elements that determine carbon allocation between source and sink tissues and that govern carbon flux along pathways for synthesis of different carbohydrate and oil sinks. It is also imperative that we gain insight into how environmental factors influence carbon partitioning [4,20]. Several proteins have been implicated as important players in carbon partitioning in plants. They include proteins involved in sugar transport and metabolism, such as sucrose transporters [21], sucrose invertases [22] and sucrose synthases [23,24], and in hexose metabolism and transport, such as hexose kinases [25] and monosaccharide transporters [26]. Other examples include proteins controlling the flux in polysaccharide biosynthesis, such as ADP-glucose pyrophosphorylase [27], and UDP-glucose pyrophosphorylase [28,29], and regulatory proteins, such as sucrose non-fermenting-1-related protein kinase [30], trehalose-6-phosphate synthase [31], and transcription factors [12,32-35].

We are interested in identifying molecular switches in cereals that direct carbon flux to different tissues and into the specific end products. We are particularly concerned with carbon partitioning between amylose, amylopectin, oil, β-glucan and fructan in cereal seeds. For the present study, we chose a rice waxy mutant, *GM077*, which is deficient in amylose biosynthesis. We examined carbon partitioning between amylose, amylopectin, oil, β-glucan, fructan and other dietary fibers in the *GM077* background, a nearly isogenic waxy line. We constructed a suppression subtractive hybridization (SSH) cDNA library between the mutant and the corresponding wild type to identify potential candidates involved in carbon partitioning. We used qPCR to verify results from the SSH experiments and to study how gene regulation controls carbon allocation in the absence of amylose biosynthesis.

## Results

### The *GM077* rice is a waxy mutant

**Figure 1:**
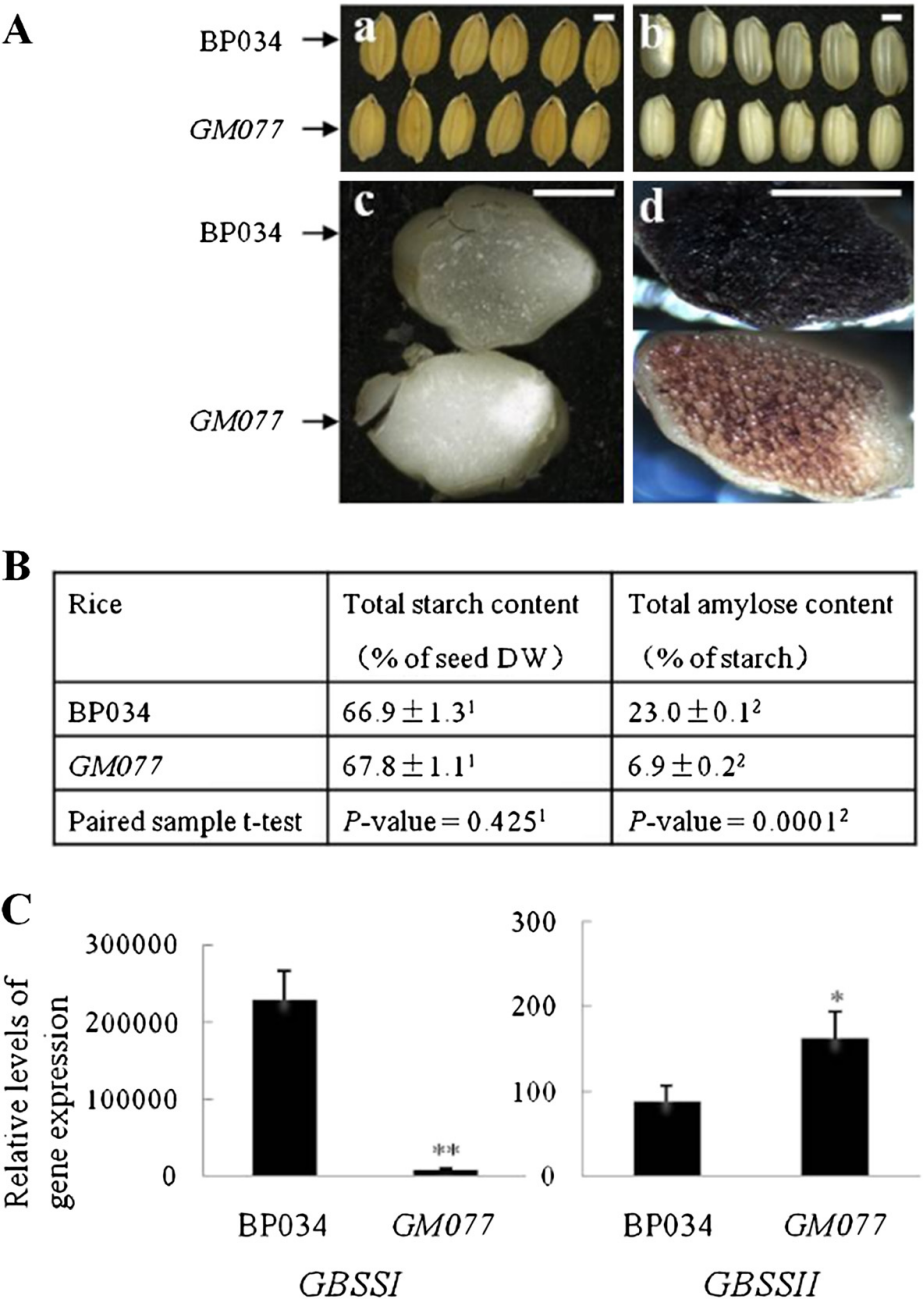
**Demonstration of *GM077 *as a waxy mutant using the corresponding wild type BP034 as a control.** (**A**) Phenotypic traits of BP034 and *GM077* grains. The grains, with and without hull (**a** and **b**, respectively), are visualized. Transverse sections of the grains without and with iodine-staining were photographed (**c** and **d**, respectively). Scale bars (=1.5 mm) are indicated. (**B**) Content of total starch and amylose was determined as Sun et al. [35]. ^1^No significant difference of total starch content between BP034 and *GM077* (*P* = 0.425). ^2^Significant difference of total amylose content between BP034 and *GM077* (*P* = 0.0001). (**C**) qPCR analysis of expression levels for *GBSSI* and *GBSSII.* DW (dry weight), GBSS (granule-bound starch synthase). The statistical difference between BP034 and *GM077* is presented as “significantly decreased” (**P < 0.01) and “increased” (*P < 0.05), respectively.

Waxy rice has been drawing much attention in rice breeding in China as it has many applications in traditional Chinese food and brewing. This has resulted in a large collection of waxy rice in the Chinese rice germplasm repositories and also in a number of breeding programs on the different qualities of waxy rice [36-38]. We selected one waxy rice cultivar, *GM077* (code No. *GM077*; Bao et al. unpublished), mainly based on the following factors: i) It is a stable mutant with a nearly isogenic background; ii) It has a relatively low amylose content (see also below) compared to other waxy mutants; iii) With the exception of its waxy grain character, *GM077* is phenotypically similar to its wild-type counterpart BP034 (code No. BP034), an elite variety of Indica rice (also cultivated under the name Guangluai No. 4 in Southern China) [38,Bao et al. unpublished] (Figure 1A-C; Additional file 1). When the grains of *GM077* were cut transversely and stained with an iodine solution, a typical reddish color of waxy starch was revealed in the endosperm [39,40]. We have further characterized the grain starch of *GM077* by recording the light absorbance of the starch-iodine complex between 200 nm and 1100 nm with a scanning spectrophotometer. We included internal standards of starch with known contents of amylose. As seen in Additional file 2, the absorbance value around 595 nm for the amylose-iodine complex was reduced proportionally with the amylose content in the starch samples, including those from the wild type (BP034) and mutant (*GM077*). Based on the absorbance, the estimated amylose content of BP034 and *GM077* is between standards 4 (26.5%) and 3 (16.2%), and standards 2 (10.4%) and 1 (1.5%), respectively. The estimations were confirmed with chemical analyses revealing a significant difference (*P* = 0.0001) in amylose content of 23.0% and 6.9% in kernels of BP034 and GMO077, respectively. The starch content was around 67% in both types of rice grains (*P* > 0.05) (Figure 1B).

It is generally accepted that amylose synthesis is carried out by granule-bound starch synthases (GBSS). Cereals have two forms of GBSS, GBSSI and GBSSII [41,42]. GBSSI is responsible for amylose synthesis in storage tissues, such as endosperm, whereas GBSSII is present in green tissues, including the pericarp of seeds. We used qPCR to analyze gene expression for both GBSS genes in rice seeds with the ubiquitin gene, *UBQ5,* as an internal standard. The qPCR results showed that, in *GM077* seeds, gene expression of *GBSSI* was significantly reduced (Figure 1C) and the expression of *UBQ5* is about the same as in the control BP034. Expression of *GBSSII* was significantly increased in *GM077* as compared with BP034.

We have shown that the *GM077* rice is a waxy mutant caused by down-regulation of *GBSSI*. Yield, kernel weight and starch content were similar between the waxy mutant and the corresponding wild type (Figure 1B; Additional file 1).To gain insight into the redistribution of carbon in the *GM077* seed, we subjected the mutant and wild-type lines to carbohydrate and oil analyses.

### The major carbon from amylose is redistributed to amylopectin in the waxy mutant

Carbohydrate analyses revealed that both *GM077* and the parental BP034 lines contained about 3% dietary fiber with similar compositions (Table 1; Additional file 3). Arabinoxylan and cellulose were major dietary fiber components (about 1% of dry caryopsis each) while mixed-linkage β-glucan and fructan were minor components. Consequently, no extra carbon was distributed into the cell walls or to the β-glucan or fructan sink in the GMO77 mutant. The starch content was slightly reduced in the waxy mutant (67.5% of dry caryopsis) compared to the wild type (69.3% of dry caryopsis) (P < 0.05). The amylose content was normal in the wild type (24% of the starch) but highly reduced in the waxy mutant (3.9% of the starch) (*P* < 0.01). Thus the amylose content in the seed was reduced from 17% of the caryopsis in the wild type to 2.6% in the waxy mutant (*P* < 0.01). The reduced content of amylose was mainly compensated for by an increased content of amylopectin in the waxy caryopsis, 65% in the waxy mutant compared to 53% in the wild type. The content of sucrose and crude oils were the same in the two rice lines (*P* > 0.05). The glucose content in the *GM077* mutant (0.2%) was somewhat higher than the wild type (0.1%) (*P* < 0.05), but was low in both lines.

**Table 1:** **Content of carbohydrates, Klason lignin and oil in BP034 and *GM077***

**Component**	**Composition**	BP034 (% of seed DW, *n *= 3*)	*GM077 *(% of seed DW, *n *= 3*)
	Rhamnose**	n.d. (not detected)	n.d.
	Fucose**	n.d.	n.d.
	Arabinose**	0.45 ± 0.08	0.43 ± 0.04
	Xylose**	0.52 ± 0.04	0.49 ± 0.07
	Mannose**	0.23 ± 0.03	0.20 ± 0.04
	Galactose**	0.13 ± 0.02	0.13 ± 0.03
	Glucose**	1.03 ± 0.13	0.92 ± 0.05
	Uronic acids**	0.26 ± 0.01	0.27 ± 0.01
	Klason lignin	0.60 ± 0.50	0.56 ± 0.44
	Fructan and fructooligosaccharides	<0.10	<0.10
**Total dietary fiber**		**3.2** ± 0.50	**3.0** ± 0.32
	β-Glucan	<0.05	<0.05
	Amylose	16.8 ± 0.70 (% of seed DW)	2.6 ± 0.17 (% of seed DW)
	Amylose	24.2 (% of starch)	3.9 (% of starch)
	Amylopectin	52.5 (% of seed DW)	64.9 (% of seed DW)
**Total starch**		**69.3** ± 0.75	**67.5** ± 0.59
**Oil**		**1.5** ± 0.21	**1.5** ± 0.21
**Free Glc**		**0.1** ± 0.00	**0.2** ± 0.00
**Free Suc**		**0.7** ± 0.15	**0.9** ± 0.15

*Mean value from three independent analytic experiments using randomly selected caryopses from a pool of six plants (see table S3). **Sugar residue.

The carbohydrate analysis thus indicated that a major fraction of carbon in the waxy mutant *GM077* was reallocated from amylose to amylopectin synthesis. This result prompted us to try to identify the genes in *GM077* responsible for this reallocation. To this end, we employed the SSH strategy (see below).

### Suppression subtractive hybridization identified 116 unigenes in the waxy mutant

**Figure 2:**
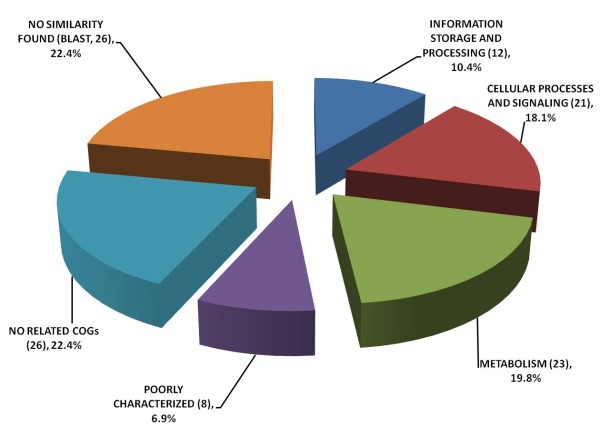
**Functional classification of the 116 unigenes from the subtractive libray of *GM077 *vs BP034.** The classification was based on BLASTX and TBLASTX results (*E*-value < 10^-5^) using the expressed sequence tags (ESTs) of the unigenes. Genes are categorized using the NCBI KOGnitor COG classification [43]. The number of unigenes in each group is indicated and their percentage in the total number of unigenes is denoted.

We used *GM077* as the tester and BP034 as the driver to construct a cDNA library after PCR amplification and SSH of cDNAs from total RNA isolated from plants at 12 days after flowering (daf). The resulting SSH library of “*GM077* vs BP034” contained 471 clones with an average length of around 500 bp. All positive clones were applied to sequencing, which returned the identification of 116 unigenes. These 116 unigenes were used for the clusters of orthologous groups (COG) functional annotation analysis [43] after BLASTX and TBLASTX against the NCBI protein databases. Among the 116 unigenes, 90 exhibited high similarity (*E*-value < 10^-5^) to known protein sequences, and 26 showed no similarity to any reported sequence. Within the 90 protein sequences, 26 lacked functional annotation. The rest of sequences were categorized in four functional groups: “information storage and processing”, “cellular processes and signaling”, “metabolism”, and “poorly characterized” (Figure 2; Additional file 4). These four functional groups have 12, 21, 23 and 8 unigenes, corresponding to 10.4%, 18.1%, 19.8% and 6.9% of the total unigenes, respectively (Figure 2). The details of the unigenes and their putative functions are shown in Table 2. Interestingly, two unigenes (clone ID No. A74 and ID No. 2B03), similar to the genes for ADP-glucose pyrophosphorylase small subunit (*AGPS*; GenBank accession No. ACJ86329.1 of the Indica group and GenBank accession No. AK103906 of the Japonica group) and isoamylase (*ISA*; GenBank accession No. BAC75533.1 of the Japonica group), respectively, were found in the carbohydrate transport and metabolism group. Notably, one clone (ID No. D25) in the group of no related COGs showed a high similarity to WRKY transcription factor 34 (GenBank accession No. NP_001060116.1 of the Japonica group). Further sequence analysis of the genes for isoamylase and WRKY transcription factor 34 revealed that they are rice orthologs to barley *ISA1* and *SUSIBA2*, respectively, previously described by Sun et al. [34,42].

**Table 2:** **Functional categories (putative functions) of proteins deduced from the obtained cDNAs (116 unigenes) after subtraction of waxy rice (*GM077*) with its wild-type (BP034)**

**Clone ID**	**Number of clones**	**Top-matched molecule in Genbank on Blastx (accession number)**	**Top-matched molecule in Genbank on Blastx (species)**	**Protein name and/or putative function**	**e-Value**	**COGs**
INFORMATION STORAGE AND PROCESSING (13)						
[J] Translation, ribosomal structure and biogenesis (3)						
D06	1	P49608.1	*Cucurbita maxima*	Aconitate hydratase, cytoplasmic	7.00E-52	KOG0452
C46	6	ACM79935.1	*Populus deltoides*	Eukaryotic translation, initiation factor 5A	7.00E-14	KOG3271
2B05	1	NP_001148134.1	*Zea mays*	Arginyl-tRNA synthetase	7.00E-62	KOG4426
[A] RNA processing and modification (NONE)						
[K] Transcription (5)						
B34	6	ACG28870.1	*Zea mays*	Transcription factor BTF3	3.00E-40	KOG2240
E14	2	BAD08114.1	*Oryza sativa*	Putative SET domain protein SDG117	1.00E-34	KOG1082
A62	8	NP_001054968.1	*Oryza sativa*	RNA polymerase I-associated factor PAF67	1.00E-39	KOG3677
C11	1	NP_001060344.1	*Oryza sativa*	Myb-related protein B (B-Myb)	7.00E-86	KOG0048
B30	1	EEE62186.1	*Oryza sativa*	Hypothetical protein OsJ_16973	7.00E-50	KOG1878
A47	2	EEE62186.1	*Oryza sativa*	Hypothetical protein OsJ_16973	1.00E-59	KOG1878
[L] Replication, recombination and repair (1)						
C54	5	EEE66658.1	*Oryza sativa*	Hypothetical protein OsJ_23285	3.00E-50	KOG4585
[B] Chromatin structure and dynamics (3)						
C41	3	NP_569031.1	*Arabidopsis thaliana*	Transducin family protein / WD-40 repeat family protein	1.00E-59	KOG1446
B86	2	NP_001047885.1	*Oryza sativa*	Nuclear protein SET domain containing protein	7.00E-121	KOG1082
A76	1	CAL54140.1	*Ostreococcus tauri*	Histones H3 and H4 (ISS)	3.00E-15	KOG1745
CELLULAR PROCESSES AND SIGNALING (21)						
[D] Cell cycle control, cell division, chromosome partitioning (2)						
D22	1	ABG65960.1	*Oryza sativa*	PAP/25A associated domain containing protein, expressed (Nucleotidyltransferase domain)	7.00E-17	KOG2277
D27	1	AAY23369.1	*Oryza sativa*	Retinoblastoma-related protein 2	4.00E-35	KOG1010
[Y] Nuclear structure (NONE)						
[V] Defense mechanisms (NONE)						
[T] Signal transduction mechanisms (3)						
B00	2	NP_194324.2	*Arabidopsis thaliana*	Epsin N-terminal homology (ENTH) domain-containing protein	1.00E-08	KOG0251
D66	1	NP_001056986.1	*Oryza sativa*	Hypothetical protein(Two-component response regulator ARR14)	5.00E-35	COG0745
E43	31	NP_001148041.1	*Zea mays*	CBL-interacting serine/threonine-protein kinase 15	1.00E-82	KOG0583
[M] Cell wall/membrane/envelope biogenesis (3)						
F70	1	AAO72599.1	*Oryza sativa*	Putative 2-dehydro-3-deoxyphosphooctonate aldolase	9.00E-66	COG2877
E73	1	AAT80327.1	*Hordeum vulgare*	UDP-D-glucuronate decarboxylase	2.00E-36	KOG1429
A21	5	AAT80327.1	*Hordeum vulgare*	UDP-D-glucuronate decarboxylase	3.00E-17	KOG1429
[N] Cell motility (NONE)						
[Z] Cytoskeleton (2)						
B38	6	NP_563908.1	*Arabidopsis thaliana*	ARK3(ARMADILLO REPEAT KINESIN 3); ATP binding/ binding/microtubule motor	1.00E-18	KOG0240
C80	11	NP_171697.3	*Arabidopsis thaliana*	Armadillo/ß-catenin repeat family protein/kinesin motor family protein	3.00E-86	KOG0240
[W] Extracellular structures (NONE)						
[U] Intracellular trafficking, secretion, and vesicular transport (4)						
F48	1	ABA95598.1	*Oryza sativa*	Clathrin heavy chain, putative, expressed	5.00E-08	KOG0985
D80	1	ABF95668.1	*Oryza sativa*	Serologically defined breastcancer antigen NY-BR-84, putative, expressed	2.00E-69	KOG2667
F23	1	ACG31280.1	*Zea mays*	ADP-ribosylation factor 1	9.00E-18	KOG0070
E39	1	NP_001150650.1	*Zea mays*	Serologically defined breast cancer antigen NY-BR-84	6.00E-32	KOG2667
[O] Posttranslational modification, protein turnover, chaperones (7)						
B28	1	AAK51086.1	*Avicennia marina*	Mitochondrial processing peptidase	2.00E-50	KOG0960
A32	1	BAB78487.1	*Oryza sativa*	26S proteasome regulatory particle non-ATPase subunit8	1.00E-21	KOG1556
C67	2	BAF00213.1	*Arabidopsis thaliana*	Polyubiquitin 4 UBQ4	5.00E-31	KOG0001
B58	1	NP_001054802.1	*Oryza sativa*	Zn-finger, RING domain containing protein	5.00E-57	KOG0800
04C04	1	ACG31834.1	*Zea mays*	Peptidyl-prolyl cis-trans isomerase NIMA-interacting 4	7.00E-38	KOG3258
D56	3	NP_001147507.1	*Zea mays*	ATP-dependent Clp protease ATP-binding subunit clpX	8.00E-08	KOG0745
D60	1	NP_001149461.1	*Zea mays*	Pyrrolidone carboxyl peptidase	9.00E-44	KOG4755
METABOLISM (23)						
[C] Energy production and conversion (5)						
D01	10	AF162665_1	*Oryza sativa*	Aldehyde dehydrogenase	5.00E-61	KOG2450
E05	3	BAB44155.1	*Bruguiera, gymnorhiza*	Hydroxypyruvate reductase	8.00E-29	KOG0069
C01	1	NP_176968.1	*Arabidopsis, thaliana*	HPR; glycerate dehydrogenase/poly(U) binding	2.00E-29	KOG0069
D14	25	ABB47885.1	*Oryza sativa*	Electron transfer flavoprotein- ubiquinone oxidoreductase, mitochondrial precursor, putative, expressed	7.00E-95	KOG2415
D33	2	NP_001149476.1	*Zea mays*	Vacuolar ATP synthase subunit F	2.00E-25	KOG3432
[G] Carbohydrate transport and metabolism (8)						
F66	1	AAA82047.1	*Oryza sativa*	Glyceraldehyde-3-phosphate dehydrogenase	2.00E-48	KOG0657
A15	1	AAO27794.1	*Gossypium hirsutum*	Glycosyl hydrolase (sugar binding domain)	6.00E-30	KOG2230
E47	22	ABG22500.1	*Oryza sativa*	Glycosyl hydrolases family 38 protein, expressed	5.00E-48	KOG1959
A64	1	ACG45298.1	*Zea mays*	Nucleotide-sugar transporter/ sugar porter	2.00E-52	KOG2234
A74	1	ACJ86329.1	*Oryza sativa*	ADP-glucose pyrophosphorylase small subunit	0.00E+00	COG0448
E73	1	AAT80327.1	*Hordeum vulgare*	UDP-D-glucuronate decarboxylase	2.00E-36	KOG1429
A21	5	AAT80327.1	*Hordeum vulgare*	UDP-D-glucuronate decarboxylase	3.00E-17	KOG1429
2B03	1	BAC75533.1	*Oryza sativa*	Isoamylase	7.00E-66	GKOG0470
[E] Amino acid transport and metabolism (3)						
E28	1	P37833.1	*Oryza sativa*	Aspartate aminotransferase, cytoplasmic	4.00E-23	KOG1411
E42	5	ACG39804.1	*Zea mays*	Histidinol-phosphate aminotransferase	2.00E-76	KOG0633
F16	1	NP_001147070.1	*Zea mays*	Nicalin	4.00E-16	KOG2526
[F] Nucleotide transport and metabolism (NONE)						
[H] Coenzyme transport and metabolism (1)						
F89	1	ACG34051.1	*Zea mays*	Farnesyl pyrophosphate synthetase	5.00E-07	KOG0711
[I] Lipid transport and metabolism (NONE)						
[P] Inorganic ion transport and metabolism (2)						
F76	1	AAP31024.1	*Oryza sativa*	Zinc transporter	7.00E-31	KOG1482
04F04	1	NP_001149686.1	*Zea mays*	Carbonic anhydrase	3.00E-13	KOG1578
[Q] Secondary metabolites biosynthesis, transport and catabolism (4)						
D58	56	AAB19117.1	*Oryza sativa*	Class III ADH enzyme	2.00E-98	KOG0022
A41	1	NP_176471.1	*Arabidopsis thaliana*	LDL1 (LSD1-LIKE1); amine oxidase/ electron carrier/ oxidoreductase	1.00E-38	KOG0029
E72	9	ACM17649.1	*Oryza rufipogon*	Alcohol dehydrogenase family-2	3.00E-25	KOG0022
C77	4	BAE00046.1	*Oryza sativa*	Alcohol dehydrogenase	4.00E-140	KOG0022
POORLY CHARACTERIZED (9)						
[R] General function prediction only (6)						
C74	1	BAB69445.1	*Oryza sativa*	Hypothetical protein	4.00E-19	KOG1901
A46	2	BAD82577.1	*Oryza sativa*	PHD finger protein-like	8.00E-13	KOG1246
D20	1	BAD11341.1	*Oryza sativa*	BRI1-KD interacting protein 113 (RNA recognition motif)	1.00E-51	KOG0118
F31	1	ABC94598.1	*Oryza sativa*	NBS-LRR type R protein, Nbs2-Pi2	1.00E-80	KOG0619
C50	8	NP_001043287.1	*Oryza sativa*	Zn-finger-like, PHD finger domain containing protein	4.00E-79	KOG1246
D53	1	EEE55043.1	*Oryza sativa*	Hypothetical protein OsJ_02730	1.00E-114	KOG0431
D42	3	EEE55043.1	*Oryza sativa*	Hypothetical protein	8.00E-93	KOG0431
[S] Function unknown (2)						
F54	1	NP_568713.1	*Arabidopsis thaliana*	Emb1879 (embryo defective 1879)	3.00E-47	KOG4249
C18	1	NP_001147117.1	*Zea mays*	Nucleotide binding protein (WD40 domain)	8.00E-32	KOG0772
NO RELATED COG (3 BeTs) (26)						
A44	11	BAD11344.1	*Oryza sativa*	BRI1-KD interacting protein 116	3.00E-36	NO RELATED
C21	2	ACN85167.1	*Oryza nivara*	MYB-CC type transfactor	5.00E-66	
C27	1	ABA95230.1	*Oryza sativa*	Retrotransposon protein, putative	9.00E-17	
F81	1	Q01881.2	*Oryza sativa*	Seed allergenic protein RA5	3.00E-08	
F15	1	AAP54389.2	*Oryza sativa*	Ulp1 protease family, C-terminal catalytic domain containing protein	7.00E-14	
F32	1	NP_001052330.1	*Oryza sativa*	Hypothetical protein	7.00E-10	
A07	2	NP_001054936.1	*Oryza sativa*	Hypothetical protein	1.00E-07	
F39	1	NP_001058150.1	*Oryza sativa*	Hypothetical protein	1.00E-41	
A43	1	NP_001066171.1	*Oryza sativa*	Conserved hypothetical protein	4.00E-07	
E29	21	EAZ06308.1	*Oryza sativa*	Hypothetical protein OsI_28542	8.00E-81	
C62	3	ABR25963.1	*Oryza sativa*	DnaJ heat shock protein	1.00E-12	
C35	9	ACA04850.1	*Picea abies*	Senescence-associated protein	8.00E-37	
B45	1	EEC77808.1	*Oryza sativa*	Hypothetical protein OsI_16996	5.00E-04	
C48	1	EEC81525.1	*Oryza sativa*	Hypothetical protein OsI_24919	1.00E-09	
D54	2	EEE68920.1	*Oryza sativa*	Hypothetical protein OsJ_27784	2.00E-100	
D79	1	NP_001149805.1	*Zea mays*	CUE domain containing protein	4.00E-06	
D25	1	NP_001060116.1	*Oryza sativa*	WRKY transcription factor 34	2.00E-72	
04C03	2	BAH91806.1	*Oryza sativa*	Conserved hypothetical protein	1.00E-04	
2B02	1	EAZ06308.1	*Oryza sativa*	Hypothetical protein OsI_28542	5.00E-54	
E36	2	CAA59142.1	*Oryza sativa*	Prolamin	4.00E-31	
B11	10	AAK13589.1	*Oryza sativa*	rRNA intron-encoded homing endonuclease	4.00E-27	
C25	1	CAA38212.1	*Oryza sativa*	Glutelin	7.00E-49	
A55	1	AAM92796.1	*Oryza sativa*	Gypothetical protein	8.00E-37	
B53	2	NP_001055525.1	*Oryza sativa*	Ubiquitin-associated domain containing protein	9.00E-54	
F77	1	EEE63701.1	*Oryza sativa*	Hypothetical protein OsJ_18519 (Ubiquitin Associated domain)	4.00E-65	
D23	1	BAD38184.1	*Oryza sativa*	C2 domain-containing protein-like	3.00E-86	
NO SIMILARITY FOUND (BLAST) (26)						

### Validation of the SHH results by semi-quantitative PCR

**Figure 3:**
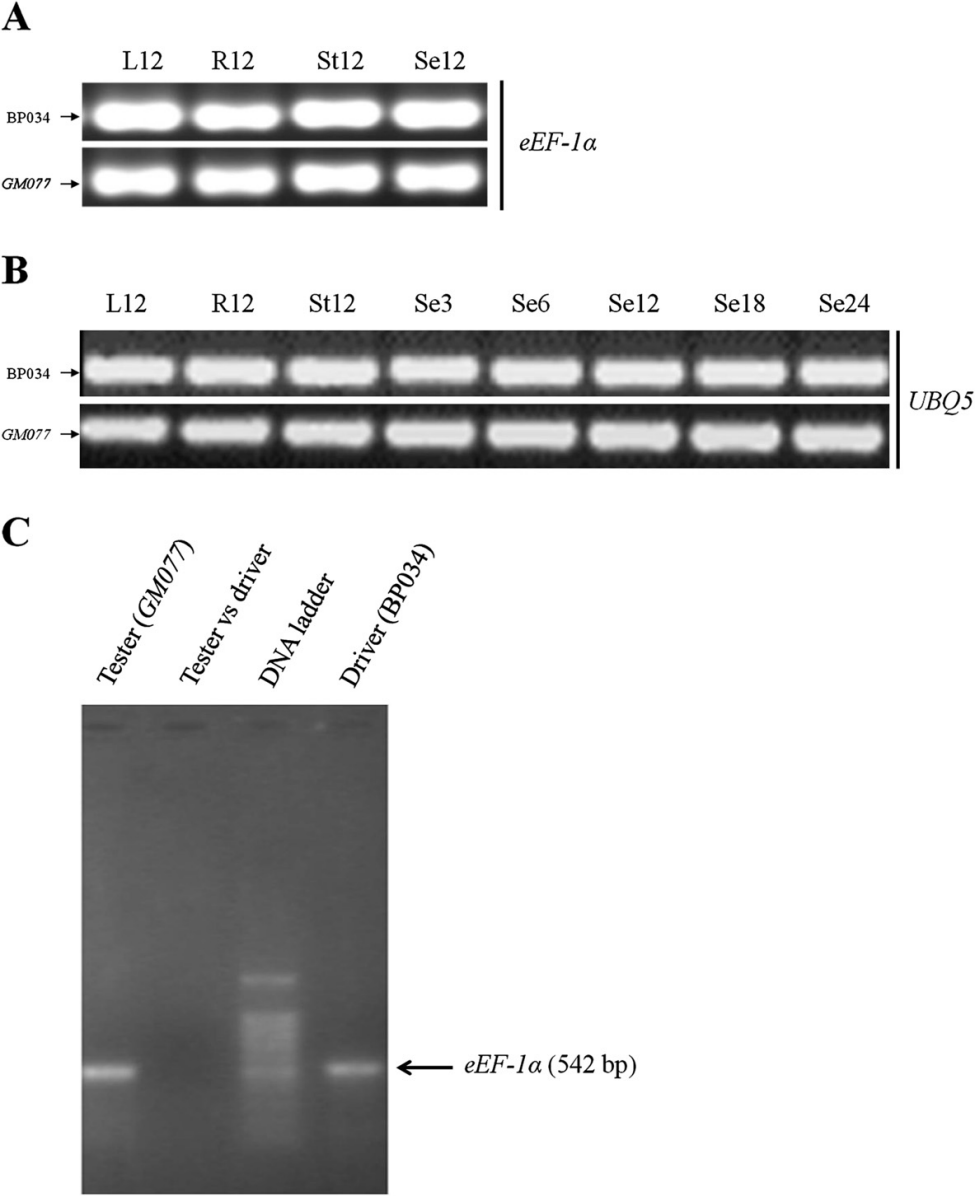
**Validation of the suppression subtractive hybridization (SSH) results by semi-quantitative RT-PCR.** (**A**) Semi-quantitative RT-PCR analysis of *eEF-1α* on the same RNA samples from BP034 and *GM077* as used in the SHH experiment, *i.e.*, RNA from seeds of 12 day after flowering (Se12), and samples from the same time point for leaves (L12), roots (R12), and stems (St12). (**B**) Semi-quantitative RT-PCR analysis of *UBQ5* on RNA samples as in the SHH experiment (Se12), and for seeds from 3, 6, 18 and 24 day after flowering, and for leaves (L12), roots (R12), and Stems (St12), respectively. (**C**) Semi-quantitative RT-PCR analysis of cDNA levels of *eEF-1α* before and after subtractive hybridization.

To verify the conclusions from the SHH experiment, we selected two housekeeping genes, the gene for the eukaryotic elongation factor-1 α subunit (*eEF-1 α*) and *UBQ5*, to follow the SSH experiment by semi-quantitative PCR. When we used the same batch of RNA as in the SHH experiment, or RNA isolated from other stages of seed development, or from other tissues, we found expression levels of the two housekeeping genes to be more or less the same in *GM077* and BP034. Furthermore, expression levels were constant throughout seed development and in different tissues of mutant and wild-type rice (Figure 3A, B). Importantly, we observed that the cDNA for *eEF-1 α* could be detected in the tester (*GM077*) and driver (BP034) samples prior to SSH, but not in the sample after subtraction hybridization (Figure 3C), lending support to the validity of the SSH approach. We also chose some additional genes, related to starch biosynthesis and carbon portioning (Materials and Methods) to further verify the reliability of the SHH experiment and to obtain detailed quantitative data on gene expression in the two rice lines. Results from those analyses are presented below.

### Gene expression profiling in the waxy mutant

**Figure 4:**
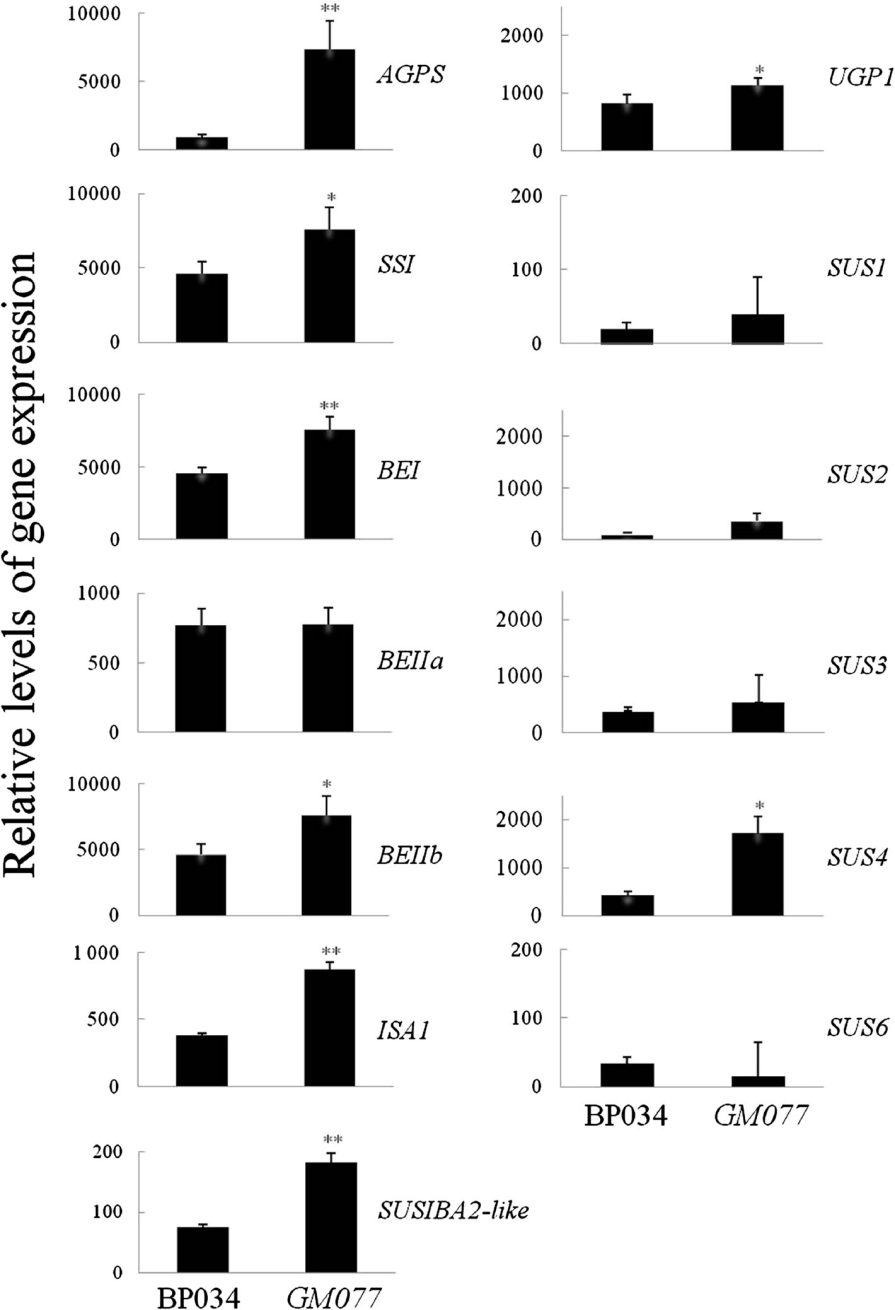
**qPCR analytic results of gene expression levels for 13 detectable genes potentially involved in carbon portioning between starch and other carbohydrates.** Biological triplets (seeds of 12 days after flowering from three different plants) and technical triplets were performed. The difference between BP034 and *GM077* was analyzed statistically by the ANOVA test and presented as “increased” (*P < 0.05) and “significantly increased” (**P < 0.01) between the two rice cultivars. Error bars are as indicated. *AGP* (gene for ADP-glucose pyrophosphorylase), *UGP* (gene for UDP-glucose pyrophosphorylase), *SS* (gene for starch synthase), *SUS* (gene for sucrose synthase), *BE* (gene for branching enzyme), *ISA* (gene for isoamylase), *SUSIBA2-like* (gene for sugar signaling in barley 2 - like).

To further validate the results from the SHH experiment and to quantify expression of genes involved in starch biosynthesis and/or carbon portioning, we chose 19 genes as representatives for gene expression analysis by qPCR, including two reference genes, *eEF-1 α* and *UBQ5* (Additional file 5). According to the results obtained by qPCR, we divided the genes into five groups (Figure 4; Table 3). The classification was based on qPCR quantification of the differential gene expression in *GM077*; “significantly decreased” (*P* < 0.01), “not changed” (*P* > 0.05), “increased” (*P* < 0.05), “significantly increased” (*P* < 0.01) and “not detected”. Intriguingly, among the four significantly increased genes, *AGPS*, *SBEI, ISA1* and *SUSIBA2-like,* all except *SBEI* were found in the SHH library. We noted that the expression level for the upregulated genes in *GM077* correlated well with the expression level for the SUSIBA2*-*like transcription factor gene (Figure 4).

**Table 3:** **Category of 19 genes with different expression levels detected by qPCR in waxy rice (*GM077*) and wild type (BP034)**

**Gene expression level (waxy/wt, or *GM077*/BP034)**	**Gene name**	**GenBank Accession No.**
Significantly decreased (*P* < 0.01)	*GBSSI*	X62134
No change (*P* > 0.05)	*BEIIa*	AB023498
	*SUS1*	OsJNBa0090P23.3
	*SUS2*	NM_001063582.1
	*SUS3*	L03366.1
	*SUS6*	OJ1149_C12-2
	*UBQ5*	AK061988
	*eEF-1α*	AK061464
Increased (*P* < 0.05)	*GBSSII*	AY069940
	*SSI*	D16202
	*BEIIb*	D16201
	*SUS4*	NM_001056599.1
	*UGP1*	DQ395328.1
Significantly increased (*P* < 0.01)	*AGPS*	AK103906
	*BEI*	D11082
	*ISA1*	AB015615
	*SUSIBA2-like*	AK121838
Not detected	*UGP2*	AF249880.1
	*SUS5/7*	OsJNBa0033H08.16/ OsJNBb0026I12.4

### Gene expression correlation of *SUSIBA2-like* and *ISA1* in the mutant and wild type

**Figure 5:**
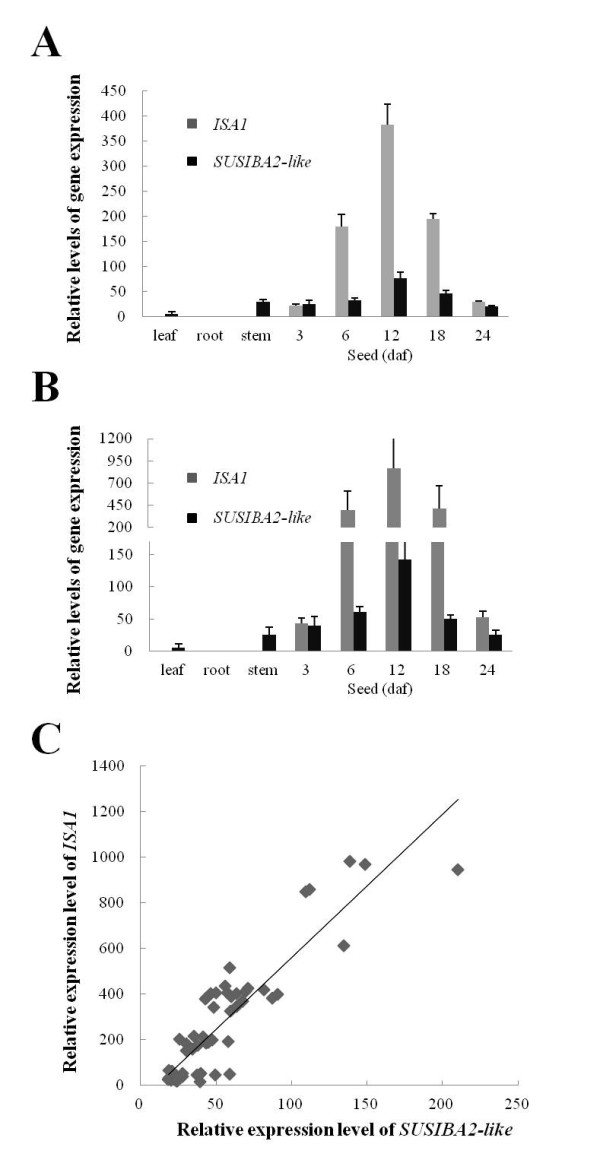
**qPCR analysis of correlation between *ISA1 *and *SUSIBA2-like *in gene expression in BP034 and *GM077. ***(**A**) Spatial and temporal expression levels of *ISA1* and *SUSIBA2-like* in the BP034 rice. (**B**) Spatial and temporal expression levels of *ISA1* and *SUSIBA2-like* in the *GM077* rice. (**C**) Plots of corresponding expression levels of *ISA1* and *SUSIBA2-like* in both BP034 and *GM077*. Statistical analysis indicated the correlation to be extremely significant (*P* < 0.01) between both *ISA1* and *SUSIBA2-like* with 0.90 of Pearson correlation coefficient (*r*). daf (days after flowering).

Sun et al. [33,34] have demonstrated that *ISA1* and *SBEIIb* in barley were upregulated by the activity of the SUSIBA2 transcription factor and a good correlation in gene expression levels has been demonstrated between *SUSIBA2* and its target genes, such as *ISA1* and *SBEIIb*[33,34,44]. To learn if this correlation holds true in rice also, and in an effort to find SUSIBA2-like-controlled genes in rice, we selected rice *ISA1* as a representative to study the correlation in expression between *SUSIBA2-like* and its target genes in rice. For this study, we chose different tissues and different time points in both the mutant *GM077* and the wild type BP034. As displayed in Figure 5A and B, there was an excellent correlation between expression levels for the two genes in the analyzed samples. The statistical analysis (Figure 5C) indicated that the relative levels of the spatial and temporal expression for the two genes in both rice lines shared a Pearson correlation coefficient (*r*) of 0.90 (*P* < 0.01).

## Discussion

Although waxy mutants of higher plants and the responsible gene (*GBSSI*) have been studied to a large extent, and the high content of amylopectin in the mutant is known [39,45-53], little information about carbon partitioning to other carbohydrates and oil fractions in waxy mutants has been reported. Moreover, gene regulation of carbon reallocation to amylopectin in the mutant is poorly understood. We are interested in the partitioning of photosynthates between starch and other storage compounds in cereal seeds. In this study, we selected one of the rice waxy mutants to follow carbon partitioning between starch and other carbohydrates when amylose biosynthesis is impeded. Our carbohydrate analysis indicated that when the amylose content is reduced, the vast majority of the assimilated carbon is reallocated to amylopectin, rather than to other carbohydrates or lipids. Interestingly, such a reallocation did not change seed weight but, rather, shifted carbon from one compound (amylose) to another (amylopectin) within the starch biosynthesis machinery.

To understand the molecular mechanisms controlling the increase in amylopectin biosynthesis, we set out to identify genes that were upregulated in the waxy mutant. From the SHH experiments we found three candidates that have previously been shown to be directly involved in starch synthesis and/or its regulation, *AGP*[54], *ISA1*[55], and *SUSIBA2-like*[34]. The functions and regulation of AGPase and isoamylase have been reviewed and well documented previously [27,45,56-59]. In cereal endosperm cells, there are two forms of AGPase, one cytosolic and one plastidic. The major fraction of ADP-glucose in cereal endosperm is believed to be produced in the cytosol and then transported to the amyloplast for subsequent starch biosynthesis. Isoamylase is suggested to play an important role in amylopectin biosynthesis and starch granule formation [45,56-58]. Both AGPase (cytosolic form) and ISA1 have been demonstrated as important players in amylopectin synthesis and starch granule formation in rice [55]. Our qPCR results indicate that AGPase S (cytosolic form) [60] and ISA1 are instrumental for the accumulation of additional amylopectin in the amylose–reduced mutant *GM077*. In the SHH experiment, we could not confirm that the identified *AGPS* corresponded to the cytosolic enzyme as the unigene sequence did not cover the transit peptide sequence region. However, our qPCR analysis of both cytosolic (Figure 4) and plastidic (not shown) forms according to Ohdan et al. [60] indicated that clone ID No. 74 should be the cytosolic form.

The mechanism behind the elevated expression of *AGP* and *ISA1* in the rice mutant remains unclear. One possibility for the enhanced *AGP* activity could be that the total amount of AGPase needs to be increased to provide ample supply of ADP-glucose when more plastidic AGPase is being recruited to multienzyme complexes for the regulation of carbon partitioning [61]. Another possibility is that the extra AGPase is required in *GM077* to convert Glc1-P to ADP-glucose in the cytosol (see also below). Since ISA1 is generally accepted as an important player in amylopectin synthesis and granule formation [45,56-58], it is not surprising that the *ISA1* expression in *GM077* significantly increased when extra amylopectin was produced in the endosperm.

We also observed that the genes for sucrose synthase 4 and UDPase 1 were upregulated in *GM077*. Since accumulation of other carbohydrates synthesized from the UDP-glucose precursor, such as cellulose and β-glucan, were unaffected in the mutant, we suggest that the increased expression of the genes for sucrose synthase 4 and UDPase 1 may be also associated with amylopectin synthesis. Sucrose synthase 4 is suggested to be cytosolic [23] and may produce UDP-glucose, which is converted by UDPase 1 to the hexose-phosphate used for amylopectin synthesis [16]. Indeed, sucrose synthases 2 and 3 in Arabidopsis, which belong to the same group as rice sucrose synthase 4 [23], have been recently reported to direct carbon to starch synthesis [62]. In our experiment, the elevated expression of cytosolic *AGP* supports that notion. Enhanced levels of AGPase may be needed to convert the Glc 1-P produced by sucrose synthase 4 and UDPase 1 to ADP-glucose for additional amylopectin synthesis. For other forms of sucrose synthases and for UDPase 2, we did not find any significant shifts in gene expression between *GM077* and BP034.

This study is centered on carbon partitioning and gene regulation in seeds of a waxy rice mutant. Our results provide no information about how carbon partitioning is regulated at the level of enzyme activity. Lü et al. [46] used transgenic rice with antisense inhibition of *GBSSI* to examine the activities of major starch synthesis enzymes. Some of the phenotypic traits observed by Lü et al. in the GMO rice were similar to what we found for the *GM077* mutant, such as no changes in seed weight and only small changes in total starch content. In accordance with our gene expression analysis, they also noticed an increase in isoamylase activities. However, they did not observe any changes in activities for AGPase or SBEs, which seems to disagree with our results at the gene activity level. We do not yet know the reason for this disparity between gene expression and enzyme activity but it should be noted that the levels of transcripts and proteins in a cell are determined by several factors, like the rate of transcription initiation, mRNA stability, efficiency of translation, and protein stability and modifications.

Our knowledge about gene regulation and the involvement of putative transcription factors in carbon partitioning is poor. Sun et al. [34,35] reported that the barley SUSIBA2 transcription factor participates in sugar signaling in barley and that it upregulates target genes by binding to the SURE-element (with an A/T rich region and a putative AAAA core) within the promoter region [34,63]. They suggested that the SURE-element(s) in promoter regions of sugar-inducible genes may play an important role in SUSIBA2-controlled gene expression. Interestingly, when we searched the promoter region of the nine upregulated genes in *GM077*, including the rice *SUSIBA2-like*, we found a number of putative SURE elements in all of the genes (Additional file 6). A very good correlation at the gene expression level was found for *SUSIBA2-like* and *ISA1*. We suggest that upregulation of *ISA1* and other genes in the *GM077* mutant is mediated by the SUSIBA2-like transcription factor. This notion is further corroborated by recent transgenic studies in rice (Hu et al. unpublished). Interestingly, when we performed a bioinformatic analysis on the gene expression patterns of the three selected genes (*SUSIBA2-like*, *ISA1* and *AGPS*) from the SSH experiment in this study using the publicly available rice and Arabidopsis microarray data, we found some correlations between *SUSIBA2-like* and the other genes (Additional file 7). However, *ISA1* is expressed in Arabidopsis leaves but not in rice leaves and the expression level of *SUSIBA2-like* is generally low in both species for reasons we do not know. Since SUSIBA2-like is a transcription factor, its gene expression level should be low. What caused the differential expression of *ISA1* in the two species is unclear. *In vitro* and *in vivo* protein-DNA interaction studies are under way to further determine the involvement of SUSIBA2-like and SURE elements in the regulation of starch biosynthesis in the rice endosperm.

In addition to their high value as starch crops, there is an increasing interest in using cereals for the production of non-starch compounds, such as β-glucan and fructan for functional foods, and oil for biofuel applications [9,10,17]. Our experimental data implicate three genes of importance for amylopectin synthesis in the rice endosperm, *AGP*, *ISA1*, and *SUSIBA2-like*. Since *AGPS* and *SUSIBA2-like* likely control the entire metabolic pathway for starch synthesis in cereals, we believe they are good targets for redirecting carbon flux from starch biosynthesis to alternative products. In fact, approaches to downregulate *AGPS* in Arabidopsis to enhance oil production at the expense of starch biosynthesis met with success [18]. It will be interesting to explore the potential for modulating *SUSIBA2* activity as a strategy for rerouting photosynthate from starch biosynthesis to other anabolic pathways in cereal seeds.

## Conclusion

Understanding of carbon allocation in cereal seeds is of great importance in plant biology. In this study we used a rice waxy mutant to gain molecular insights into how amylose deficiency affects carbon allocation in cereal seeds. Analysis of carbohydrate and oil fractions in the waxy mutant showed that when amylose is deficient, carbon is mainly allocated to amylopectin rather than to other carbon end products, such as β-glucan or oil. Gene expression profiling identified several candidate genes implicated in the carbon reallocation response. These genes included *AGP* and *SUSIBA2-like*. We suggest that these two genes are promising targets in efforts to redirect carbon flux in cereal seeds from starch biosynthesis to alternative carbon end products. To our knowledge, this study is the first comparative analysis of carbon fractions and gene expression profiling on a global scale in a waxy mutant.

## Methods

### Plant materials and growth

Rice seeds of the BP034 and *GM077* cultivars were obtained from the waxy rice-breeding program at the Institute of Nuclear Agricultural Sciences, Zhejiang University, China. The *GM077* mutant was originally generated by γ-irradiation in the waxy rice-breeding program [36-38, Bao et al. unpublished]. It has been developed to a nearly isogenic background through many years of breeding. The rice plants were field-grown on the campus farm at Zhejiang University. Individual tillers were labeled at flowering. Seed samples were harvested on day 3, 6, 12, 18 and 24 after flowering, respectively. At least 6 panicles from different individuals of BP034 or *GM077* were sampled at each time point. At the same time points (day 3, 6, 12, 18 and 24 after flowering), the leaves, stems and roots of the corresponding rice plants were harvested. The harvested tissues were immediately frozen in liquid nitrogen and kept at -80°C until use.

### Carbohydrate analyses

Mature and dry seeds were prepared as described previously [44,64,65]. Iodine staining and spectrophotometer scanning were performed as described by Sun et al. [35]. Total starch and amylose contents were pre-analyzed as described by Sun et al. [35]. Dietary fiber components were analyzed with the Uppsala method [66] and fructan (including fructooligosaccharides) as described by Rakha et al. [67]. Total dietary fiber was calculated as the sum of fiber components analyzed with the Uppsala method and fructan. The mixed-linkage β-glucan content was analyzed as described by McCleary and Codd [64], the starch content as described by Santacruz et al. [68] and the amylose content as described by Chrastil [69]. The arabinoxylan content was calculated as the sum of arabinose and xylose residues determined by the Uppsala method, the cellulose content as the difference between glucose residues determined by the Uppsala method and the mixed-linkage β-glucan content, and the amylopectin content as the difference between the starch and amylose contents. Free Glc and Suc were analyzed according to Bergmeyer et al. [70] and Bernt and Bergmeyer [71], respectively. The crude oil content was determined according to the European standard method [72].

### Oligonucleotides

Oligonucleotides used in the experiments for qPCR, semi-quantitative PCR, and SHH are listed in Additional file 5. Nineteen representative genes were selected including the two reference genes *eEF-1α* and *UBQ5*. The oligonucleotides were purchased from Invitrogen (Carlsbad, CA, USA).

### RNA isolation

Total RNA was isolated according to the protocol described previously [34,35].

### Quantitative PCR (qPCR) and semi-quantitative PCR

qPCR and semi-quantitative PCR were performed as described previously [34,73]. The SYBR Green Master Mix and cDNA synthesis kit were purchased from Toyobo (Osaka, Japan) and Promega (Madison, WI, USA), respectively. A real-time PCR machine, iQ5 from Bio-Rad (Hercules, CA, USA), was used for qPCR and a PCR thermo cycler, MJ Research PTC-200 (GMI, Ramsey, MN, USA), was used for semi-quantitative PCR. The rice genes of *eEF-1α* and *UBQ5* were used as endogenous references for data normalization [74] in qPCR. The relative transcript level was calculated by the method of 2^-ΔCt^[74].

### Construction of a cDNA subtractive library of *GM077* vs BP034

The cDNA subtractive library of *GM077* vs BP034 was constructed using the SSH technique [75]. Total RNA of *GM077* from seeds at 12 daf was used as the tester and the corresponding sample of BP034 as the driver. The protocol in Dai et al. [76] was followed with the following modifications: i) Transcripts were enriched by *in vitro* transcription; and ii) Duplex-specific nuclease (DSN)-mediated normalization and subtraction were used. The procedure is outlined in Additional file 8, and all linkers, adapters and PCR primers are listed in Additional file 5. PCR products generated by SHH were digested by *Sal*I and cloned in the pUC19 vector. Recombinant plasmids were used to transform *Escherichia coli* DH5α. Transformed bacteria were applied to LB plates containing 50 μg ml^-1^ ampicillin for selection and 40 μg ml^-1^ X-gal for detection of α-complementation [77]. White and positive colonies were picked for colony PCR screening to check inserts. Positive colonies with inserts were propagated. Plasmids were isolated and sequenced at Beijing Genomics Institute (BGI, Beijing, China) using the M13 forward and reverse primers. The *eEF-1α* gene was used to monitor efficiency of the suppression subtractive hybridization by semi-quantitative PCR.

### Bioinformatics and statistical analysis

The obtained sequences were edited by the DNAstar® software (Madison, WI, USA). Unigene sequences were used for BLASTX and TBLASTX searches against the protein database ( http://blast.ncbi.nlm.nih.gov/). The retrieved proteins with high sequence similarities (*E*-value < 10^-5^) were categorized using the NCBI KOGnitor COG classification ( http://www.ncbi.nlm.nih.gov/COG) based on the method of Tatusov et al. [43]. The *cis*-element analysis of gene promoters was performed using the BioEdit software (Carlsbad, CA, US). The significance of differences in obtained data was tested by ANOVA (analysis of variance) with a threshold *P*-value of 0.05 ( http://www.ats.ucla.edu/stat/). Publicly available microarray data for rice ( http://ricexpro.dna.affrc.go.jp) and for Arabidopsis ( http://www.weigelworld.org/resources/microarray/AtGenExpress) were used for bioinformatics analyses of gene expression patterns of *SUSIBA2-like*, *ISA1* and *AGPS*.

## Competing interests

The authors declare that they have no competing interests.

## Authors’ contributions

M-ZZ and J-HF did the experiments of SSH, semi-quantitative and qPCR, starch pre-analysis and bioinformatics analysis. XY carried out statistical and promoter analysis, and partial bioinformatics analysis. JL did partially the experiment of qPCR. J-SB did the breeding work in many years to generate the nearly isogenic waxy mutant, *GM077*. GF performed the carbohydrate and oil analysis. RA, CJ and PÅ were involved in the carbohydrate and oil analysis and in revising the manuscript. CS contributed to the experimental design, coordination of the study, drafting the manuscript and interpreting the results. All authors read and approved the final manuscript.

## Supplementary Material

Additional file 1 **Phenotypic traits of BP034 and *GM077.*** Click here for file

Additional file 2 **Absorbance spectra of the iodine-stained starch samples from BP034 and *GM077. ***Starch standard samples with known amylose contents are included in the spectra. ST (standard), AC (amylose content). The iodine-staining was performed as described previously [35]. Click here for file

Additional file 3 **Content of carbohydrates, Klason lignin and oil in BP034 and *GM077.*** Click here for file

Additional file 4 **Functional categories in Clusters of Orthologous Groups (COGs) for proteins deduced from the obtained cDNAs after subtraction of *GM077 *(tester) with BP034 (driver).** Click here for file

Additional file 5 Oligonucleotides. Click here for file

Additional file 6 **Putative SURE-elements in promoter regions of the upregulated genes indentified in *GM077. ***GenBank accession number for each gene is listed in Table 3. The putative SURE-element sequence (in green) was based on Sun et al. [34] & Grierson et al. [63]. The nucleotide position is relative to translation initiate site (the ATG codon). *GBSS* (gene for granule-bound starch synthase), *AGP* (gene for ADP-glucose pyrophosphorylase), *SS* (gene for starch synthase), *BE* (gene for branching enzyme), *ISA* (gene for isoamylase), *SUSIBA2-like* (gene for sugar signaling in barley 2-like), *UGP* (gene for UDP-glucose pyrophosphorylase), *SUS* (gene for sucrose synthase). Click here for file

Additional file 7 **Gene expression profiling of three selected genes (*SUSIBA2-like, ISA1 *and *AGPS*) from the SSH experiment during plant development of rice and Arabidopsis.** The microarray data from two publicly available websites was used for rice ( http://ricexpro.dna.affrc.go.jp) and Arabidopsis ( http://www.weigelworld.org/resources/microarray/AtGenExpress), respectively. (A) Rice *SUSIBA2-like* (GenBank Ac No. AK121838). (B) Arabidopsis *WRKY20* (a homologue of *SUSIBA2*, GenBank Ac No. NM_11898). (C) Rice *ISA1* (GenBank Ac No. AB015615). (D) Arabidopsis *ISA1* (GenBank Ac No. NM_128551). (E) Rice *AGPS* (GenBank Ac No. AK103906). (F) Arabidopsis *AGPS* (GenBank Ac No. NM_124205). Click here for file

Additional file 8 **A flow chart of DSN-mediated (duplex-specific nuclease) suppression subtractive hybridization (SSH).** A small amount of RNA samples from tester (*GM077*) and driver (BP034) was used for template-switching cDNA synthesis and step-out PCR amplification [78]. SP6 and T7 RNA polymerases were then employed to generate sufficient tester and driver transcripts, respectively. After a secondary reverse transcription and RNA digestion, the tester cDNAs were subjected to an excess amount of driver RNA for hybridization. Hybridization was performed by denaturation and ressociation. cDNAs in hybrids with RNA were digested by duplex-specific nuclease. The left-over single-stranded cDNAs from hybridization were only the temples for exponential PCR amplification to generate cDNA fragments for construction of a cDNA library. Tsp (template-switching primer), 3’ap (adaptor primer), PI (primer I). Click here for file

## References

[B1] Charalampopoulos D Wang R Pandiella SS Webb C Application of cereals and cereal components in functional foods Int J Food Microbiol 2002 79 131 141 12382693 10.1016/s0168-1605(02)00187-3

[B2] Tharanathan RN Food-derived carbohydrates–structural complexity and functional diversity Crit Rev Biotechnol 2002 22 65 84 11958336 10.1080/07388550290789469

[B3] Banas A Debski H Banas W Heneen WK Dahlqvist A Bafor M Gummeson PO Marttila S Ekman A Carlsson AS Stymne S Lipids in grain tissues of oat (Avena sativa): differences in content, time of deposition, and fatty acid composition J Exp Bot 2007 58 2463 2470 17586606 10.1093/jxb/erm125

[B4] Geigenberger P Regulation of starch biosynthesis in response to a fluctuating environment Plant Physiol 2011 155 1566 1577 21378102 10.1104/pp.110.170399 PMC3091114

[B5] Blennow A Engelsen SB Helix-breaking news: fighting crystalline starch energy deposits in the cell Trends Plant Sci 2010 15 236 240 20149714 10.1016/j.tplants.2010.01.009

[B6] Kamal-Eldin A Lærke HN Knudsen KE Lampi AM Piironen V Adlercreutz H Katina K Poutanen K Åman P Physical, microscopic and chemical characterisation of industrial rye and wheat brans from the Nordic countries Food Nutr Res 2009 10.3402/fnr.v53i0.1912 PMC2675562 19412350

[B7] Jones PJ Dietary agents that target gastrointestinal and hepatic handling of bile acids and cholesterol J Clin Lipidol 2008 2 S4 10 21291720 10.1016/j.jacl.2008.01.005

[B8] Andersson AA Lampi AM Nyström L Piironen V Li L Ward JL Gebruers K Courtin CM Delcour JA Boros D Fraś A Dynkowska W Rakszegi M Bedo Z Shewry PR Åman P Phytochemical and dietary fiber components in barley varieties in the HEALTHGRAIN Diversity Screen J Agric Food Chem 2008 56 9767 9776 18921979 10.1021/jf802037f

[B9] Hayden DM Rolletschek H Borisjuk L Corwin J Kliebenstein DJ Grimberg A Stymne S Dehesh K Cofactome analyses reveal enhanced flux of carbon into oil for potential biofuel production Plant J 2011 67 1018 1028 21615570 10.1111/j.1365-313X.2011.04654.x

[B10] Nalawade S Nalawade S Liu C Jansson C Sun C Development of an efficient tissue culture after crossing (TCC) system for transgenic improvement of barley as a bioenergy crop Appl Energy 2012 91 405 411

[B11] Pouvreau B Baud S Vernoud V Morin V Py C Gendrot G Pichon JP Rouster J Paul W Rogowsky PM Duplicate maize Wrinkled1 transcription factors activate target genes involved in seed oil biosynthesis Plant Physiol 2011 156 674 686 21474435 10.1104/pp.111.173641 PMC3177267

[B12] Shen B Allen WB Zheng P Li C Glassman K Ranch J Nubel D Tarczynski MC Expression of ZmLEC1 and ZmWRI1 increases seed oil production in maize Plant Physiol 2010 153 980 987 20488892 10.1104/pp.110.157537 PMC2899924

[B13] Alonso AP Val DL Shachar-Hill Y Central metabolic fluxes in the endosperm of developing maize seeds and their implications for metabolic engineering Metab Eng 2011 13 96 107 20969971 10.1016/j.ymben.2010.10.002

[B14] Ayre BG Membrane-transport systems for sucrose in relation to whole-plant carbon partitioning Mol Plant 2011 4 377 394 21502663 10.1093/mp/ssr014

[B15] Roitsch T Source-sink regulation by sugar and stress Curr Opin Plant Biol 1999 2 198 206 10375568 10.1016/S1369-5266(99)80036-3

[B16] Emes MJ Bowsher CG Hedley C Burrell MM Scrase-Field ES Tetlow IJ Starch synthesis and carbon partitioning in developing endosperm J Exp Bot 2003 54 569 575 12508067 10.1093/jxb/erg089

[B17] Ekman A Hayden DM Dehesh K Bülow L Stymne S Carbon partitioning between oil and carbohydrates in developing oat (Avena sativa L.) seeds J Exp Bot 2008 59 4247 4257 19036843 10.1093/jxb/ern266 PMC2639027

[B18] Sanjaya Durrett TP Weise SE Benning C Increasing the energy density of vegetative tissues by diverting carbon from starch to oil biosynthesis in transgenic Arabidopsis Plant Biotechnol J 2011 9 874 883 22003502 10.1111/j.1467-7652.2011.00599.x

[B19] Gout E Bligny R Douce R Boisson AM Rivasseau C Early response of plant cell to carbon deprivation: in vivo 31P-NMR spectroscopy shows a quasi-instantaneous disruption on cytosolic sugars, phosphorylated intermediates of energy metabolism, phosphate partitioning, and intracellular pHs New Phytol 2011 189 135 147 20819175 10.1111/j.1469-8137.2010.03449.x

[B20] Geigenberger P Kolbe A Tiessen A Redox regulation of carbon storage and partitioning in response to light and sugars J Exp Bot 2005 56 1469 1479 15863446 10.1093/jxb/eri178

[B21] Kühn C Grof CP Sucrose transporters of higher plants Curr Opin Plant Biol 2010 13 288 298 20303321 10.1016/j.pbi.2010.02.001

[B22] Ruan YL Jin Y Yang YJ Li GJ Boyer JS Sugar input, metabolism, and signaling mediated by invertase: roles in development, yield potential, and response to drought and heat Mol Plant 2010 3 942 955 20729475 10.1093/mp/ssq044

[B23] Cho JI Kim HB Kim CY Hahn TR Jeon JS Identification and characterization of the duplicate rice sucrose synthase genes OsSUS5 and OsSUS7 which are associated with the plasma membrane Mol Cells 2011 31 553 561 21533550 10.1007/s10059-011-1038-y PMC3887615

[B24] Jansson C Westerbergh A Zhang J Hu X Sun C Cassava, a potential biofuel crop in (the ) People’s Republic of China Appl Energy 2009 86 S95 S99

[B25] Halford NG Paul MJ Carbon metabolite sensing and signaling Plant Biotechnol J 2003 1 381 398 17134398 10.1046/j.1467-7652.2003.00046.x

[B26] Slewinski TL Diverse functional roles of monosaccharide transporters and their homologs in vascular plants: a physiological perspective Mol Plant 2011 4 641 662 21746702 10.1093/mp/ssr051

[B27] Comparot-Moss S Denyer K The evolution of the starch biosynthetic pathway in cereals and other grasses J Exp Bot 2009 60 2481 2492 19505928 10.1093/jxb/erp141

[B28] Kleczkowski LA Geisler M Fitzek E Wilczynska M A common structural blueprint for plant UDP-sugar-producing pyrophosphorylases Biochem J 2011 439 375 379 21992098 10.1042/BJ20110730

[B29] Park JI Ishimizu T Suwabe K Sudo K Masuko H Hakozaki H Nou IS Suzuki G Watanabe M UDP-glucose pyrophosphorylase is rate limiting in vegetative and reproductive phases in Arabidopsis thaliana Plant Cell Physiol 2010 51 981 996 20435647 10.1093/pcp/pcq057

[B30] Ghillebert R Swinnen E Wen J Vandesteene L Ramon M Norga K Rolland F Winderickx J The AMPK/SNF1/SnRK1 fuel gauge and energy regulator: structure, function and regulation FEBS J 2011 278 3978 3990 21883929 10.1111/j.1742-4658.2011.08315.x

[B31] Eastmond PJ Graham IA Trehalose metabolism: a regulatory role for trehalose-6-phosphate? Curr Opin Plant Biol 2003 6 231 235 12753972 10.1016/s1369-5266(03)00037-2

[B32] Weselake RJ Taylor DC Rahman MH Shah S Laroche A McVetty PB Harwood JL Increasing the flow of carbon into seed oil Biotechnol Adv 2009 27 866 878 19625012 10.1016/j.biotechadv.2009.07.001

[B33] Shi L Katavic V Yu Y Kunst L Haughn G Arabidopsis glabra2 mutant seeds deficient in mucilage biosynthesis produce more oil Plant J 2012 69 37 46 21883555 10.1111/j.1365-313X.2011.04768.x

[B34] Sun C Palmqvist S Olsson H Borén M Ahlandsberg S Jansson C A novel WRKY transcription factor, SUSIBA2, participates in sugar signaling in barley by binding to the sugar-responsive elements of the iso1 promoter Plant Cell 2003 15 2076 2092 12953112 10.1105/tpc.014597 PMC181332

[B35] Sun C Höglund AS Olsson H Mangelsen E Jansson C Antisense oligodeoxynucleotide inhibition as a potent strategy in plant biology: identification of SUSIBA2 as a transcriptional activator in plant sugar signalling Plant J 2005 44 128 138 16167901 10.1111/j.1365-313X.2005.02515.x

[B36] Bao JS Corke H Sun M Genetic diversity in the physicochemical properties of waxy rice (Oryza sativa L.) starch J Sci Food Agric 2004 84 1299 1306

[B37] Bao JS Corke H Sun M Analysis of genetic diversity and relationship in Genetic diversity in waxy rice (Oryza sativa L.) using AFLP and ISSR markers Genet Resour Crop Evol2006a 53 323 330

[B38] Bao JS Corke H Sun M Nucleotide diversity in starch synthase IIa and validation of single nucleotide polymorphisms in relation to starch gelatinization temperature and other physicochemical properties in rice (Oryza sativa L.) Theor Appl Genet2006b 113 1171 1183 16850313 10.1007/s00122-006-0355-6

[B39] Terada R Nakajima M Isshiki M Okagaki RJ Wessler SR Shimamoto K Antisense waxy genes with highly active promoters effectively suppress waxy gene expression in transgenic rice Plant Cell Physiol 2000 41 881 888 10965945 10.1093/pcp/pcd008

[B40] Nakamura T Yamamori M Hirano H Hidaka S Nagamine T Production of waxy (amylose-free) wheats Mol Gen Genet 1995 248 253 259 7565586 10.1007/BF02191591

[B41] Vrinten PL Nakamura T Wheat granule-bound starch synthase I and II are encoded by separate genes that are expressed in different tissues Plant Physiol 2000 122 255 264 10631269 10.1104/pp.122.1.255 PMC58864

[B42] Hirose T Terao T A comprehensive expression analysis of the starch synthase gene family in rice (Oryza sativa L.) Planta 2004 220 9 16 15232694 10.1007/s00425-004-1314-6

[B43] Tatusov RL Fedorova ND Jackson JD Jacobs AR Kiryutin B Koonin EV Krylov DM Mazumder R Mekhedov SL Nikolskaya AN Rao BS Smirnov S Sverdlov AV Vasudevan S Wolf YI Yin JJ Natale DA The COG database: an updated version includes eukaryotes BMC Bioinforma 2003 4 41 10.1186/1471-2105-4-41 PMC222959 12969510

[B44] Sun C Sathish P Ahlandsberg S Jansson C Analyses of isoamylase gene activity in wild-type barley indicate its involvement in starch synthesis Plant Mol Biol 1999 40 431 443 10437827 10.1023/a:1006217506090

[B45] Tian Z Qian Q Liu Q Yan M Liu X Yan C Liu G Gao Z Tang S Zeng D Wang Y Yu J Gu M Li J Allelic diversities in rice starch biosynthesis lead to a diverse array of rice eating and cooking qualities Proc Natl Acad Sci USA 2009 106 21760 21765 20018713 10.1073/pnas.0912396106 PMC2793318

[B46] Lü B Guo Z Liang J Effects of the activities of key enzymes involved in starch biosynthesis on the fine structure of amylopectin in developing rice (Oryza sativa L.) endosperms Sci China C Life Sci 2008 51 863 871 18815749 10.1007/s11427-008-0120-y

[B47] Itoh K Ozaki H Okada K Hori H Takeda Y Mitsui T Introduction of Wx transgene into rice wx mutants leads to both high- and low-amylose rice Plant Cell Physiol 2003 44 473 480 12773633 10.1093/pcp/pcg068

[B48] Sato Y Nishio T Mutation detection in rice waxy mutants by PCR-RF-SSCP Theor Appl Genet 2003 107 560 567 12734656 10.1007/s00122-003-1282-4

[B49] Patron NJ Smith AM Fahy BF Hylton CM Naldrett MJ Rossnagel BG Denyer K The altered pattern of amylose accumulation in the endosperm of low-amylose barley cultivars is attributable to a single mutant allele of granule-bound starch synthase I with a deletion in the 5'-non-coding region Plant Physiol 2002 130 190 198 12226499 10.1104/pp.005454 PMC166552

[B50] Fujita N Hasegawa H Taira T The isolation and characterization of a waxy mutant of diploid wheat (Triticum monococcum L.) Plant Sci 2001 160 595 602 11448734 10.1016/s0168-9452(00)00408-8

[B51] Vrinten P Nakamura T Yamamori M Molecular characterization of waxy mutations in wheat Mol Gene Genet 1999 261 463 471 10.1007/s004380050989 10323226

[B52] Okagaki RJ Neuffer MG Wessler SR A deletion common to two independently derived waxy mutations of maize Genetics 1991 128 425 431 2071021 10.1093/genetics/128.2.425 PMC1204479

[B53] Okagaki RJ Wessler SR Comparison of non-mutant and mutant waxy genes in rice and maize Genetics 1988 120 1137 1143 2906308 10.1093/genetics/120.4.1137 PMC1203576

[B54] Lee SK Hwang SK Han M Eom JS Kang HG Han Y Choi SB Cho MH Bhoo SH An G Hahn TR Okita TW Jeon JS Identification of the ADP-glucose pyrophosphorylase isoforms essential for starch synthesis in the leaf and seed endosperm of rice (Oryza sativa L.) Plant Mol Biol 2007 65 531 546 17406793 10.1007/s11103-007-9153-z

[B55] Kawagoe Y Kubo A Satoh H Takaiwa F Nakamura Y Roles of isoamylase and ADP-glucose pyrophosphorylase in starch granule synthesis in rice endosperm Plant J 2005 42 164 174 15807780 10.1111/j.1365-313X.2005.02367.x

[B56] Ball S Colleoni C Cenci U Raj WK Tirtiaux C The evolution of glycogen and starch metabolism in eukaryotes gives molecular clues to understand the establishment of plastid endosymbiosis J Exp Bot 2011 62 1775 1801 21220783 10.1093/jxb/erq411

[B57] Zeeman SC Kossmann J Smith AM Starch: its metabolism, evolution, and biotechnological modification in plants Ann Rev Plant Biol 2010 61 209 234 20192737 10.1146/annurev-arplant-042809-112301

[B58] Jeon JS Ryoo N Hahn TR Walia H Nakamura Y Starch biosynthesis in cereal endosperm Plant Physiol Biochem 2010 48 383 392 20400324 10.1016/j.plaphy.2010.03.006

[B59] Hannah LC James M The complexities of starch biosynthesis in cereal endosperms Curr Opin Biotechnol 2008 19 160 165 18400487 10.1016/j.copbio.2008.02.013

[B60] Ohdan T Francisco PB Jr Sawada T Hirose T Terao T Satoh H Nakamura Y Expression profiling of genes involved in starch synthesis in sink and source organs of rice J Exp Bot 2005 56 3229 3244 16275672 10.1093/jxb/eri292

[B61] Hennen-Bierwagen TA Lin Q Grimaud F Planchot V Keeling PL James MG Myers AM Proteins from multiple metabolic pathways associate with starch biosynthetic enzymes in high molecular weight complexes: a model for regulation of carbon allocation in maize amyloplasts Plant Physiol 2009 149 1541 1559 19168640 10.1104/pp.109.135293 PMC2649383

[B62] Angeles-Núñez JG Tiessen A Arabidopsis sucrose synthase 2 and 3 modulate metabolic homeostasis and direct carbon towards starch synthesis in developing seeds Planta 2010 232 701 718 20559653 10.1007/s00425-010-1207-9

[B63] Grierson C Du JS de Torres Zabala M Beggs K Smith C Holdsworth M Bevan M Separate cis sequences and trans factors direct metabolic and developmental regulation of a potato tuber storage protein gene Plant J 1994 5 815 826 8054988 10.1046/j.1365-313x.1994.5060815.x

[B64] McCleary BV Codd R Measurement of (1→3), (1→4)-β-D-glucan in barley and oats: a streamlined enzymic procedure J Sci Food Agric 1991 55 303 312

[B65] Sun C Sathish P Ahlandsberg S Jansson C The two genes encoding starch-branching enzymes IIa and IIb are differentially expressed in barley Plant Physiol 1998 118 37 49 9733524 10.1104/pp.118.1.37 PMC34872

[B66] Theander O Aman P Westerlund E Andersson R Pettersson D Total dietary fiber determined as neutral sugar residues, uronic acid residues, and Klason lignin (the Uppsala method): collaborative study J AOAC Int 1995 78 1030 1044 7580315

[B67] Rakha A Åman P Andersson R Characterization of dietary fibre components in rye products Food Chem 2010 119 859 867

[B68] Santacruz S Koch K Andersson R Åman P Characterization of potato leaf starch J Agric Food Chem 2004 52 1985 1989 15053540 10.1021/jf030601k

[B69] Chrastil J Improved colorimetric determination of amylose in starches or flours Carbohydr Res 1987 159 154 158

[B70] Bergmeyer HU Bernt E Schmidt F Stork H Methods of Enzymatic Analysis (Bergmeyer HU, ed.) 1974 3 New York and London: Verlag Chemie, Weinheim/Academic Press, Inc 1196 1201

[B71] Bernt E Bergmeyer HU Methods of Enzymatic Analysis (Bergmeyer HU, ed.) 1974 3 New York and London: Verlag Chemie, Weinheim/Academic Press, Inc 1304 1207

[B72] Anonymous Determination of crude oils and fat (Method B) Off J Eur Communities 1984 15 29 30

[B73] Mangelsen E Wanke D Kilian J Sundberg E Harter K Jansson C Significance of light, sugar, and amino acid supply for diurnal gene regulation in developing barley caryopses Plant Physiol 2010 153 14 33 20304969 10.1104/pp.110.154856 PMC2862414

[B74] Jain M Nijhawan A Tyagi AK Khurana JP Validation of housekeeping genes as internal control for studying gene expression in rice by quantitative real-time PCR Biochem Biophys Res Commun 2006 345 646 651 16690022 10.1016/j.bbrc.2006.04.140

[B75] Diatchenko L Lau YF Campbell AP Chenchik A Moqadam F Huang B Lukyanov S Lukyanov K Gurskaya N Sverdlov ED Siebert PD Suppression subtractive hybridization: a method for generating differentially regulated or tissue-specific cDNA probes and libraries Proc Natl Acad Sci USA 1996 93 6025 6030 8650213 10.1073/pnas.93.12.6025 PMC39182

[B76] Dai ZM Zhu XJ Yang WJ Full-length normalization subtractive hybridization: a novel method for generating differentially expressed cDNAs Mol Biotechnol 2009 43 257 263 19669953 10.1007/s12033-009-9198-0

[B77] Sambrook J Fritsch EF Maniatis T Molecular cloning: a laboratory manual 1989 Cold Spring Harbor, NY: Cold Spring Harbor Laboratory Press

[B78] Matz M Shagin D Bogdanova E Britanova O Lukyanov S Diatchenko L Chenchik A Amplification of cDNA ends based on template-switching effect and step-out PCR Nucleic Acids Res 1999 27 1558 1560 10037822 10.1093/nar/27.6.1558 PMC148354

