# Supplementary material for: Molecular insights into how a deficiency of amylose affects carbon allocation – carbohydrate and oil analyses and gene expression profiling in the seeds of a rice waxy mutant
Source: BMC Plant Biol. 2012 Dec 5;12:230. doi: 10.1186/1471-2229-12-230 (PMC3541260; doi:10.1186/1471-2229-12-230)
Supplement: Additional file 1 — Phenotypic traits of BP034 and GM077. [file 1471-2229-12-230-S1.docx]

**Additional file 1** Phenotypic traits of BP034 and *GM077*

| Phenotypic trait | BP034 | *GM077* |
| --- | --- | --- |
| Heading date (days after sowing) | 62.3 ± 0.6 | 62.0 ± 1.0 |
| Plant height (cm) | 75.3 ± 2.6 | 74.0 ± 0.8 |
| Flag leaf length (cm) | 26.6 ± 1.4 | 26.4 ± 1.4 |
| Flag leaf width (cm) | 1.8 ± 0.1 | 1.8 ± 0.1 |
| Grain length (mm) | 7.5 ± 0.1 | 7.5 ± 0.1 |
| Grain width (mm) | 3.2 ± 0.0 | 3.2 ± 0.0 |
| Panicle length (cm) | 20.1 ± 0.7 | 20.2 ± 0.9 |
| Thousand-grain weight (g) | 18.9 ± 0.2 | 19.4 ± 0.1 |

No significant difference between BP034 and *GM077* (*P* > 0.05)
