# Supplementary figures and images for: Molecular insights into how a deficiency of amylose affects carbon allocation – carbohydrate and oil analyses and gene expression profiling in the seeds of a rice waxy mutant
Source: BMC Plant Biol. 2012 Dec 5;12:230. doi: 10.1186/1471-2229-12-230 (PMC3541260; doi:10.1186/1471-2229-12-230)

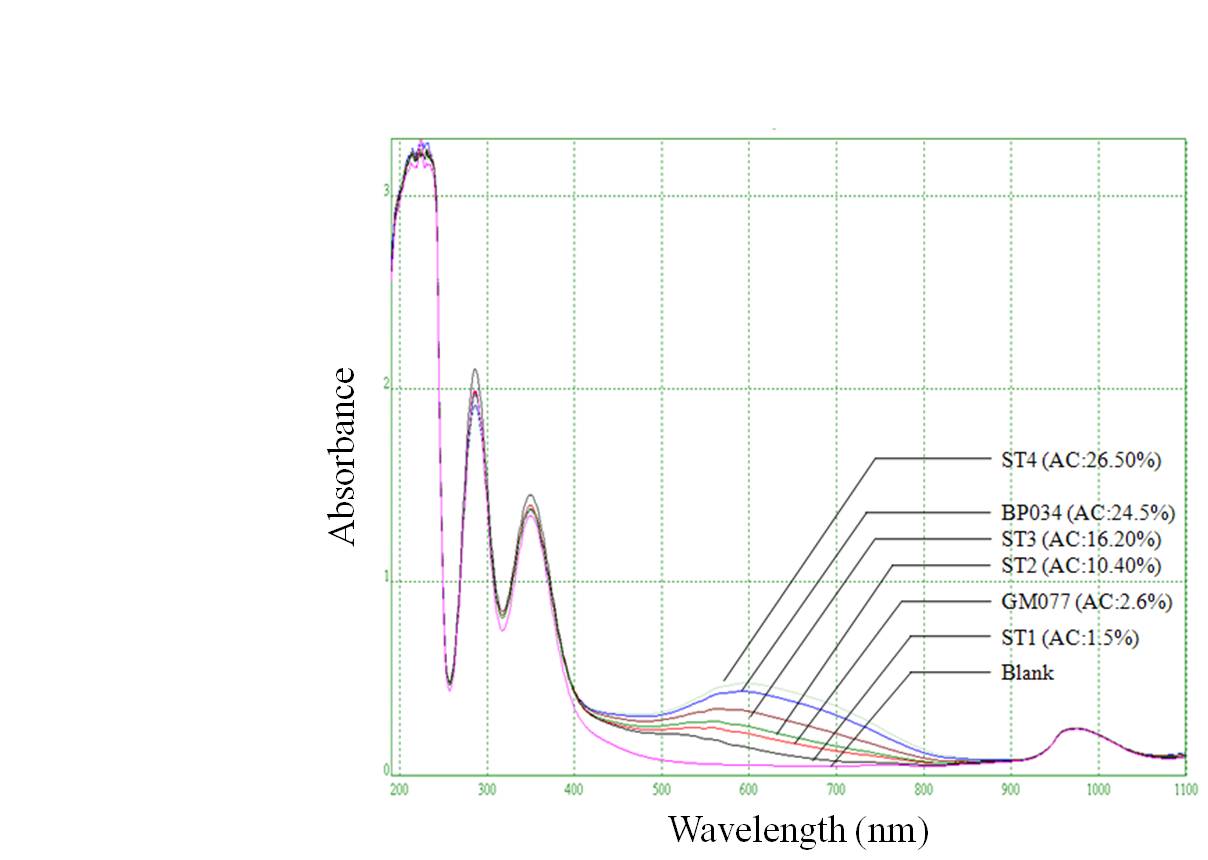

Supplement: Additional file 2 — Absorbance spectra of the iodine-stained starch samples from BP034 and GM077. Starch standard samples with known amylose contents are included in the spectra. ST (standard), AC (amylose content). The iodine-staining was performed as described previously [35]. [file 1471-2229-12-230-S2.jpeg]

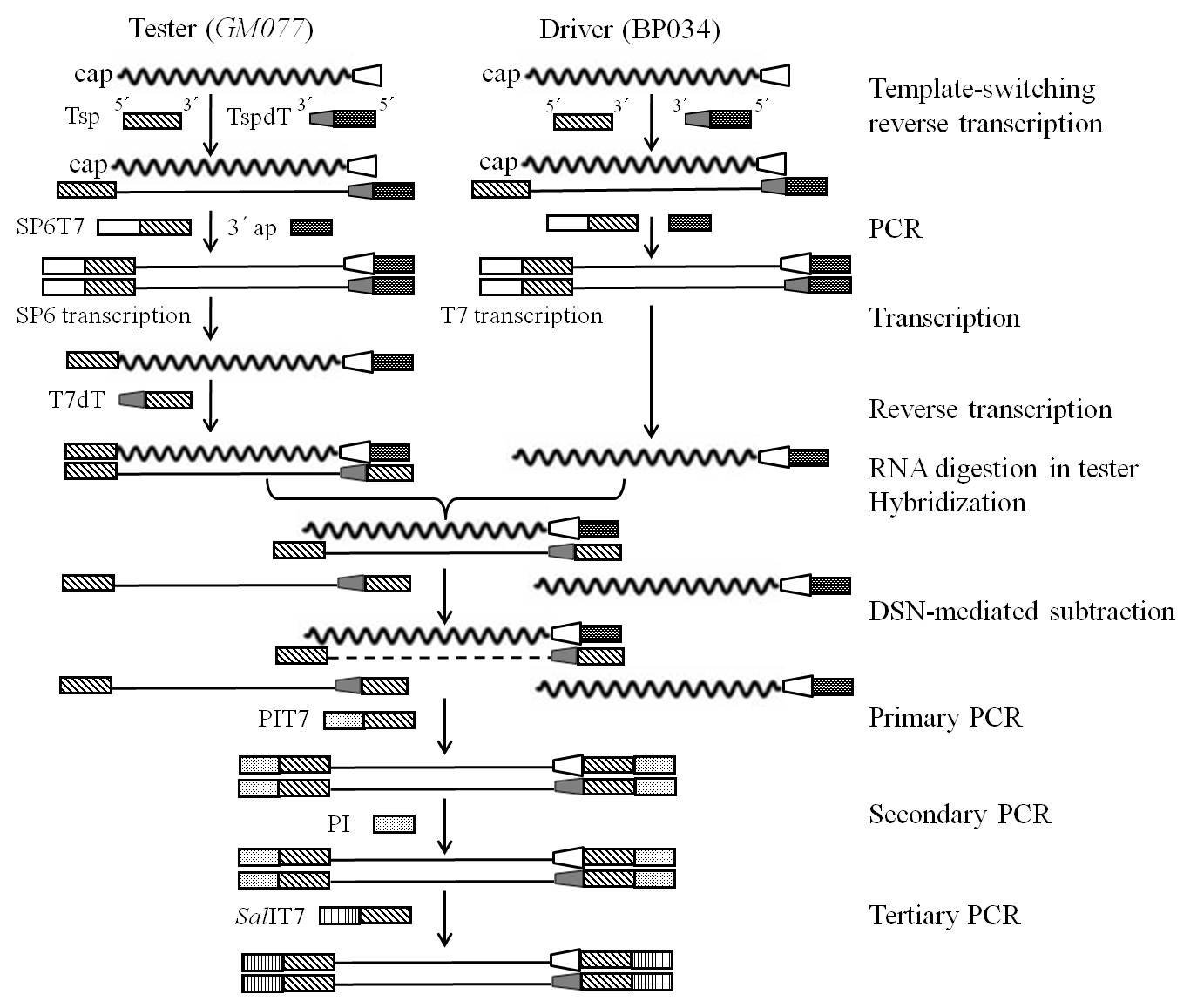

Supplement: Additional file 7 — Gene expression profiling of three selected genes (SUSIBA2-like, ISA1 and AGPS) from the SSH experiment during plant development of rice and Arabidopsis. The microarray data from two publicly available websites was used for rice ( http://ricexpro.dna.affrc.go.jp) and Arabidopsis ( http://www.weigelworld.org/resources/microarray/AtGenExpress), respectively. (A) Rice SUSIBA2-like (GenBank Ac No. AK121838). (B) Arabidopsis WRKY20 (a homologue of SUSIBA2, GenBank Ac No. NM_11898). (C) Rice ISA1 (GenBank Ac No. AB015615). (D) Arabidopsis ISA1 (GenBank Ac No. NM_128551). (E) Rice AGPS (GenBank Ac No. AK103906). (F) Arabidopsis AGPS (GenBank Ac No. NM_124205). [file 1471-2229-12-230-S7.jpeg]

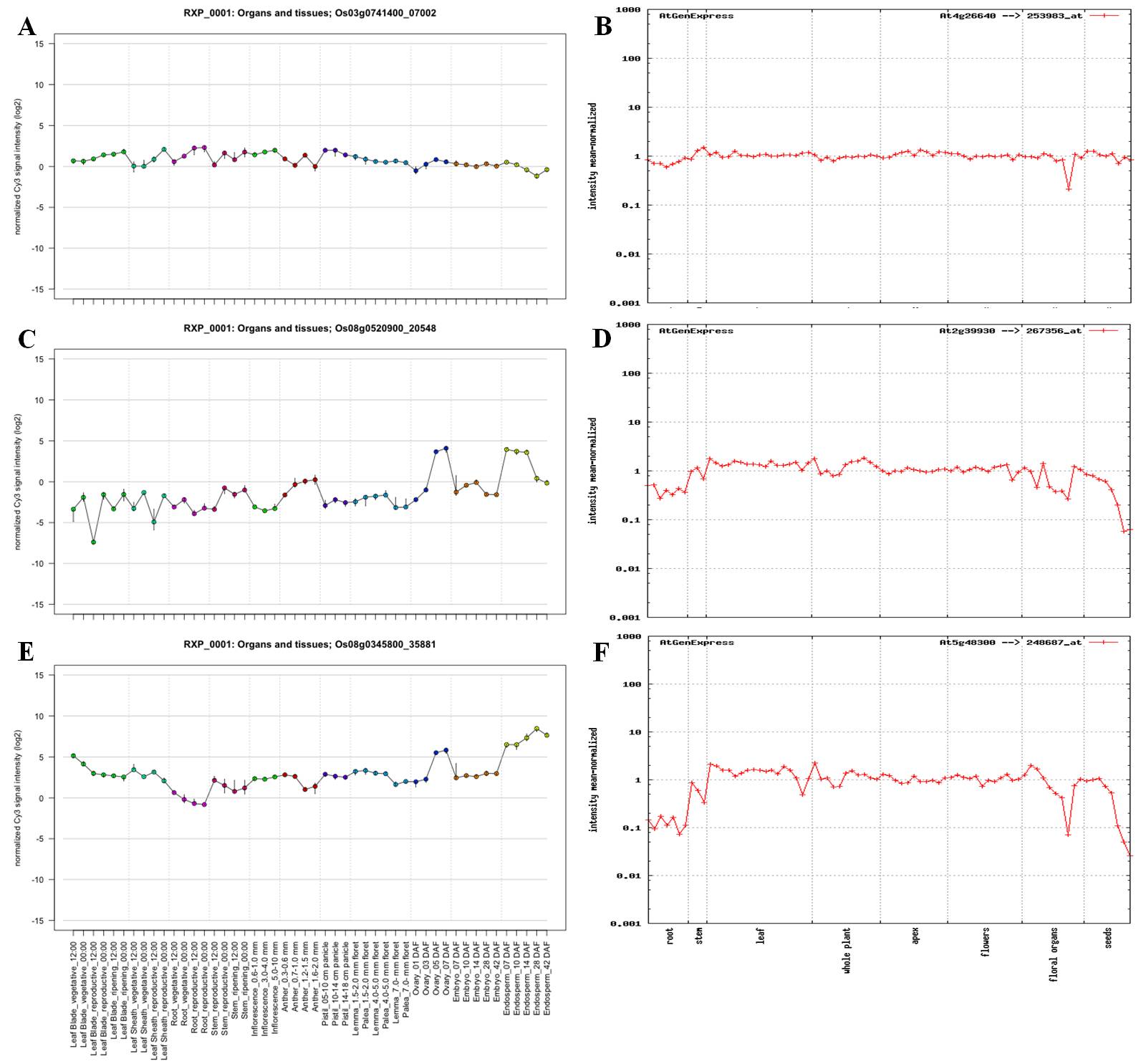

Supplement: Additional file 8 — A flow chart of DSN-mediated (duplex-specific nuclease) suppression subtractive hybridization (SSH). A small amount of RNA samples from tester (GM077) and driver (BP034) was used for template-switching cDNA synthesis and step-out PCR amplification [78]. SP6 and T7 RNA polymerases were then employed to generate sufficient tester and driver transcripts, respectively. After a secondary reverse transcription and RNA digestion, the tester cDNAs were subjected to an excess amount of driver RNA for hybridization. Hybridization was performed by denaturation and ressociation. cDNAs in hybrids with RNA were digested by duplex-specific nuclease. The left-over single-stranded cDNAs from hybridization were only the temples for exponential PCR amplification to generate cDNA fragments for construction of a cDNA library. Tsp (template-switching primer), 3’ap (adaptor primer), PI (primer I). [file 1471-2229-12-230-S8.jpeg]
