# Supplementary material for: Molecular insights into how a deficiency of amylose affects carbon allocation – carbohydrate and oil analyses and gene expression profiling in the seeds of a rice waxy mutant
Source: BMC Plant Biol. 2012 Dec 5;12:230. doi: 10.1186/1471-2229-12-230 (PMC3541260; doi:10.1186/1471-2229-12-230)
Supplement: Additional file 3 — Content of carbohydrates, Klason lignin and oil in BP034 and GM077. [file 1471-2229-12-230-S3.docx]

**Additional file 3** Content of carbohydrates, Klason lignin and oil in BP034 and *GM077*

| Component | BP034 |  |  | Average |  | *GM077* |  |  | Average | *P*-value (ANOVA test) between BP034 and *GM077* |
| --- | --- | --- | --- | --- | --- | --- | --- | --- | --- | --- |
|  | a | b | c |  |  | a | b | c |  |  |
| Rhamnose^1^ | n.d. | n.d. | n.d | n.d. |  | n.d. | n.d. | n.d. | n.d. |  |
| Fucose^1^ | n.d. | n.d. | n.d | n.d. |  | n.d. | n.d. | n.d. | n.d. |  |
| Arabinose^1^ | 0.54 | 0.43 | 0.39 | 0.45 ± 0.08 |  | 0.48 | 0.42 | 0.40 | 0.43 ± 0.04 | 0.71 |
| Xylose^1^ | 0.56 | 0.48 | 0.51 | 0.52 ± 0.04 |  | 0.56 | 0.50 | 0.42 | 0.49 ± 0.07 | 0.69 |
| Mannose^1^ | 0.26 | 0.20 | 0.23 | 0.23 ± 0.03 |  | 0.17 | 0.19 | 0.24 | 0.20 ± 0.04 | 0.33 |
| Galactose^1^ | 0.15 | 0.14 | 0.11 | 0.13 ± 0.02 |  | 0.12 | 0.17 | 0.11 | 0.13 ± 0.03 | 1.00 |
| Glucose^1^ | 1.15 | 0.89 | 1.05 | 1.03 ± 0.13 |  | 0.94 | 0.95 | 0.86 | 0.92 ± 0.05 | 0.24 |
| Uronic acids^1^ | 0.25 | 0.25 | 0.27 | 0.26 ± 0.01 |  | 0.27 | 0.27 | 0.26 | 0.27 ± 0.01 | 0.42 |
| Klason lignin | 0.30 | 0.33 | 1.18 | 0.60 ± 0.50 |  | 0.31 | 0.30 | 1.07 | 0.56 ± 0.44 | 0.92 |
| Fructan and fructooligosaccharides |  |  |  | <0.10 |  |  |  |  | <0.10 |  |
|  |  |  |  |  |  |  |  |  |  |  |
| Total DF | 3.2 | 2.7 | 3.7 | 3.2 ± 0.50 |  | 2.9 | 2.8 | 3.4 | 3.0 ± 0.32 | 0.55 |
| β Glucan |  |  |  | <0.05 |  |  |  |  | <0.05 |  |
|  |  |  |  |  |  |  |  |  |  |  |
| Starch | 70.0 | 69.3 | 68.5 | 69.3 ± 0.75 |  | 67.9 | 67.7 | 66.8 | 67.5 ± 0.59 | 0.03 |
| Amylose | 17.6 | 16.5 | 16.3 | 16.8 ± 0.70 |  | 2.8 | 2.5 | 2.5 | 2.6 ± 0.17 | 0.00 |
| Amylose (% of starch) |  |  |  | 24.2 |  |  |  |  | 3.9 |  |
| Amylopectin |  |  |  | 52.5 |  |  |  |  | 64.9 |  |
|  |  |  |  |  |  |  |  |  |  |  |
| Oil | 1.3 | 1.7 | 1.4 | 1.5 ± 0.21 |  | 1.7 | 1.3 | 1.6 | 1.5 ± 0.21 | 0.87 |
| Free Glc | 0.1 | 0.1 | 0.1 | 0.1 ± 0.00 |  | 0.2 | 0.2 | 0.2 | 0.2 ± 0.00 | 0.05 |
| Free Suc | 0.5 | 0.7 | 0.8 | 0.7 ± 0.15 |  | 0.9 | 0.8 | 1.1 | 0.9 ± 0.15 | 0.09 |

Data from three independently technical analyses. Seeds from a pool of six plants were randomly selected and prepared according to Materials and Methods. ^1^Sugar residue
