# Supplementary material for: Molecular insights into how a deficiency of amylose affects carbon allocation – carbohydrate and oil analyses and gene expression profiling in the seeds of a rice waxy mutant
Source: BMC Plant Biol. 2012 Dec 5;12:230. doi: 10.1186/1471-2229-12-230 (PMC3541260; doi:10.1186/1471-2229-12-230)
Supplement: Additional file 4 — Functional categories in Clusters of Orthologous Groups (COGs) for proteins deduced from the obtained cDNAs after subtraction of GM077 (tester) with BP034 (driver). [file 1471-2229-12-230-S4.docx]

**Additional file 4** Functional categories in Clusters of Orthologous Groups (COGs) for proteins deduced from the obtained cDNAs after subtraction of *GM077* (tester) with BP034 (driver)

| Functional category | Number of obtained cDNAs |
| --- | --- |
| **INFORMATION STORAGE AND PROCESSING** | **12** |
| [J] Translation, ribosomal structure and biogenesis | 3 |
| [A] RNA processing and modification | 0 |
| [K] Transcription | 5 |
| [L] Replication, recombination and repair | 1 |
| [B] Chromatin structure and dynamics | 3 |
| **CELLULAR PROCESSES AND SIGNALING** | **21** |
| [D] Cell cycle control, cell division, chromosome partitioning | 2 |
| [Y] Nuclear structure | 0 |
| [V] Defense mechanisms | 0 |
| [T] Signal transduction mechanisms | 3 |
| [M] Cell wall/membrane/envelope biogenesis | 3 |
| [N] Cell motility | 0 |
| [Z] Cytoskeleton | 2 |
| [W] Extracellular structures | 0 |
| [U] Intracellular trafficking, secretion, and vesicular transport | 4 |
| [O] Posttranslational modification, protein turnover, chaperones | 7 |
| **METABOLISM** | **23** |
| [C] Energy production and conversion | 5 |
| [G] Carbohydrate transport and metabolism | 8 |
| [E] Amino acid transport and metabolism | 3 |
| [F] Nucleotide transport and metabolism | 0 |
| [H] Coenzyme transport and metabolism | 1 |
| [I] Lipid transport and metabolism | 0 |
| [P] Inorganic ion transport and metabolism | 2 |
| [Q] Secondary metabolites biosynthesis, transport and catabolism | 4 |
| **POORLY CHARACTERIZED** | **8** |
| [R] General function prediction only | 6 |
| [S] Function unknown | 2 |
| **NO RELATED COGs** | **26** |
| **NO SIMILARITY FOUND (BLAST)** | **26** |
