# Supplementary material for: Molecular insights into how a deficiency of amylose affects carbon allocation – carbohydrate and oil analyses and gene expression profiling in the seeds of a rice waxy mutant
Source: BMC Plant Biol. 2012 Dec 5;12:230. doi: 10.1186/1471-2229-12-230 (PMC3541260; doi:10.1186/1471-2229-12-230)
Supplement: Additional file 5 — Oligonucleotides. [file 1471-2229-12-230-S5.docx]

**Additional file 5** Oligonucleotides

| Oligonucleotides for quantitative and semi-quantitative PCR | | | |
| --- | --- | --- | --- |
| Gene name | | GenBank Accession No | Sequence (5´→ 3´) |
| *GBSSI* | | X62134 | F: AACGTGGCTGCTCCTTGAA  R: TTGGCAATAAGCCACACACA |
| *GBSSII* | | AY069940 | F: AGGCATCGAGGGTGAGGAG  R: CCATCTGGCCCACATCTCTA |
| *AGPS* | | AK103906 | F: AACAATCGAAGCGCGAGAAA  R: GCCTGTAGTTGGCACCCAGA |
| *SSI* | | D16202 | F: GGGCCTTCATGGATCAACC  R: CCGCTTCAAGCATCCTCATC |
| *BEI* | | D11082 | F: TGGCCATGGAAGAGTTGGC  R: CAGAAGCAACTGCTCCACC |
| *BEIIa* | | AB023498 | F: GCCAATGCCAGGAAGATGA  R: GCGCAACATAGGATGGGTTT |
| *BEIIb* | | D16201 | F: ATGCTAGAGTTTGACCGC  R: AGTGTGATGGATCCTGCC |
| *ISA1* | | AB015615 | F: CGGGTACAGGTTCGATGGTATGTT  R: GAGGTAGGTCACCTTGCCAATCAA |
| *SUSIBA2-like* | | AK121838 | F: AACCGTGAGCCTCGTGTTGTAGT  R: GACTGGGCATCCTGTATTTGTGC |
| *UGP1* | | DQ395328.1 | F: TGGAGCAGCCATTCGGTTCTTT  R: ATGGGTTTGTTCTGGCTGGGTT |
| *UGP2* | | AF249880.1 | F: TGAAGCAGCGGCAAGAACACAA  R: TTTGCCCATTCCACATGCTCCT |
| *SUS1* | | OsJNBa0090P23.3 | F: AGGGACATCATGCAATCAGCGT  R: ACCAGCAGCATCCTCTGCAAAT |
| *SUS2* | | NM_001063582.1 | F: ACAAGGGCACGACAATGATGCT  R: AAGCACACGCTTTGCACAGTCA |
| *SUS3* | | L03366.1 | F: ACCAATGAGCTTGTGGCTGTCT  R: TTGCGGCGTTGTACTCAGCAAT |
| *SUS4* | | NM_001056599.1 | F: AGGGACATCATGCAATCAGCGT  R: ACCAGCAGCATCCTCTGCAAAT |
| *SUS5/7* | | OsJNBa0033H08.16/  OsJNBb0026I12.4 | F: GATTCAGATGTTAAGTGGAGAGAG  R: ATCTTGTTTATCTCCTCGATCTC |
| *SUS6* | | OJ1149_C12-2 | F: GGGTGACACTGCTGAAACATGCAA  R: ACCTTCTCCTGGCCAAAGTAACCA |
| *UBQ5* | | AK061988 | F: ACCACTTCGACCGCCACTACT  R: ACGCCTAAGCCTGCTGGTT |
| *eEF-1α* | | AK061464 | F: TTTCACTCTTGGTGTGAAGCAGAT  R: GACTTCCTTCACGATTTCATCGTAA |
| Oligonucleotides for the SSH experiment | | | |
| Oligonucleotide name | Sequence (5´→ 3´)* | | |
| Tsp | **GTAATACGACTCACTATAGGG**GG | | |
| TspdT | TGGTTGGACTCGGTTTGGACGCCATAGAATTGG(T)_15_ | | |
| SP6T7 | CATTTAGGTGACACTATAGA**GTAATACGACTCACTATAGGG** | | |
| 3’ap | TGGTTGGACTCGGTTTGGACG | | |
| T7dT | **GTAATACGACTCACTATAGGG**GG(T)_15_ | | |
| PIT7 | CTGCAGCGAACCAATCCTCT**GTAATACGACTCACTATAGGG** | | |
| PI | CTGCAGCGAACCAATCCTCTG | | |
| *Sal*IT7 | GATCGTCGAC**GTAATACGACTCACTATAGGG** | | |
| T7 | **GTAATACGACTCACTATAGGG** | | |
| eEF-1αF | GGCTACAACCCTGACAAGATTC | | |
| eEF-1αR | TCAGCAAACTTGACGGCAATGT | | |

*T7 promoter sequence is in bold and the sequence of 3´ap is underlined
