# Supplementary material for: Molecular insights into how a deficiency of amylose affects carbon allocation – carbohydrate and oil analyses and gene expression profiling in the seeds of a rice waxy mutant
Source: BMC Plant Biol. 2012 Dec 5;12:230. doi: 10.1186/1471-2229-12-230 (PMC3541260; doi:10.1186/1471-2229-12-230)
Supplement: Additional file 6 — Putative SURE-elements in promoter regions of the upregulated genes indentified in GM077. GenBank accession number for each gene is listed in Table 3. The putative SURE-element sequence (in green) was based on Sun et al. [34] & Grierson et al. [63]. The nucleotide position is relative to translation initiate site (the ATG codon). GBSS (gene for granule-bound starch synthase), AGP (gene for ADP-glucose pyrophosphorylase), SS (gene for starch synthase), BE (gene for branching enzyme), ISA (gene for isoamylase), SUSIBA2-like (gene for sugar signaling in barley 2-like), UGP (gene for UDP-glucose pyrophosphorylase), SUS (gene for sucrose synthase). [file 1471-2229-12-230-S6.docx]

|  |  | 10 20 30 40 |  |
| --- | --- | --- | --- |
| **Position** | **Gene name** | ....\|....\|....\|....\|....\|....\|....\|....\|....\|.. | **Position** |
| -999 | ***NST*** | **---TATGGTCTAAATGTTTCATAAAACACCATCTTTATTTAAA----** | -960 |
| -953 | ***NST*** | **-----TTGAATTCAATATATCTAAAACAATATATTTTAAAATGGTA-** | -913 |
| -941 | ***NST*** | **--TATCTAAAACAATATATTTTAAAATGGTAAATAGTTAAACC----** | -901 |
| -904 | ***NST*** | **--GTTTAACTATTTACCATTTTAAAATATATTGTTTTAGATAT----** | -944 |
| -857 | ***NST*** | **--ACGTTATATATTTTATGTTTAAAATTTTTAGTAAATACTTG----** | -817 |
| -826 | ***NST*** | **--TTACTAAAAATTTTAAACATAAAATATATAACGTGTATATG----** | -866 |
| -820 | ***NST*** | **--CTTGACAACAACTCCCTCCGAAAAAAAAGGAGATAAACTCT----** | -780 |
| -811 | ***NST*** | **--GTTGTTGTCAAGTATTTACTAAAAATTTTAAACATAAAATA----** | -851 |
| -724 | ***NST*** | **--TACTCCATCCGTTCTTAAAGAAAAAAAAAGTAAACTCTAGC----** | -764 |
| -709 | ***NST*** | **--TATAAGGGTGGGGGGTTTTCAAAACTTTAAAGTGTTAGGTG----** | -669 |
| -673 | ***NST*** | **--CCTAACACTTTAAAGTTTTGAAAACCCCCCACCCTTATATA----** | -713 |
| -625 | ***NST*** | **--TTATGATGCAAGTTGCATATAAAACCACAGGTATTATAACC----** | -665 |
| -565 | ***NST*** | **--ATTATTTGCTCGGTTCGTTCAAAATAGAGCGAAATTTCGAT----** | -605 |
| -837 | ***GBSSII*** | **-AATCATAGACTAATTAGGCTCAAAAATTCGTCTCGCGATTT-----** | -797 |
| -756 | ***GBSSII*** | **-TATTTGATGTGATGTTTTTGGAAAAAAAATTTAAACTAAAT-----** | -716 |
| -634 | ***GBSSII*** | **-TTAAACTAAATCAGAGGCCTTAAAATAAAAGAGTTAG---------** | -594 |
| -532 | ***GBSSII*** | **--TAAATCAGAGGCCTTAAAATAAAAGAGTTAGGATATCCG------** | -492 |
| -506 | ***GBSSII*** | **-CGGCTGCTTGTACGTGCTGACAAAAAAAAAAGAACTTGGAA-----** | -466 |
| -501 | ***GBSSII*** | **-AGGGGTTTTCAAACACGAATGAAAAAACTAATTTCATAGCT-----** | -541 |
| -498 | ***GBSSII*** | **-AAACCACACACCACCCGCACGAAAAAAACCGAACCGCACGT-----** | -458 |
| -498 | ***GBSSII*** | **CACGTTAGTGAAATCGCTCTCTAAAACTGTCAAGGAAGTTA------** | -538 |
| -469 | ***GBSSII*** | **----AACTAAATCAGAGGCCTTAAAATAAAAGAGTTAGGATATCC--** | -509 |
| -366 | ***GBSSII*** | **-TTAAACTAAATCAGAGGCCTTAAAATAAAAGAGTTAGGATA-----** | -406 |
| -244 | ***GBSSII*** | **--ATTTGATGTGATGTTTTTGGAAAAAAAATTTAAACTAAATC----** | -284 |
| -162 | ***GBSSII*** | **-AATCATAGACTAATTAGGCTCAAAAATTCGTCTCGCGATTT-----** | -202 |
| -740 | ***AGPS2b*** | **ATTTTTTAGGCATGCTGTTATTAAAATATAAAATAGTTCTT------** | -700 |
| -734 | ***AGPS2b*** | **------TAGGCATGCTGTTATTAAAATATAAAATAGTTCTTGACTAT** | -694 |
| -714 | ***AGPS2b*** | **-TATTTTAATAACAGCATGCCTAAAAAATAATTCTAGAATTC-----** | -754 |
| -597 | ***AGPS2b*** | **AAGTGGTTAAATATAGAAGGGTAAAATTATATATGTAAGGA------** | -637 |
| -545 | ***AGPS2b*** | **-CTATCACGCCACATGCCATAGAAAAACTTGGATTTAAATAC-----** | -585 |
| -496 | ***AGPS2b*** | **AATAACCACATATTATTGTTCTAAAAAAACCACATATTTTA------** | -456 |
| -495 | ***AGPS2b*** | **-ATAACCACATATTATTGTTCTAAAAAAACCACATATTTTAT-----** | -455 |
| -436 | ***AGPS2b*** | **CATGACACATACTTGCACATATAAAATATGTGGTTTTTTTA------** | -476 |
| -224 | ***AGPS2b*** | **--TACAAAAGAATCTCTTTTTGAAAAAACTAAAATTACAACAA----** | -184 |
| -218 | ***AGPS2b*** | **AGAATCTCTTTTTGAAAAAACTAAAATTACAACAAAAACGG------** | -178 |
| -186 | ***AGPS2b*** | **-TGTTGTAATTTTAGTTTTTTCAAAAAGAGATTCTTTTGTAG-----** | -226 |
| -944 | ***SSI*** | **-ATAACAGGTAGGGAATTCACTAAAAACAAAGTTCAATAGAA-----** | -984 |
| -861 | ***SSI*** | **TTTTTTTCGCTTTTTTCTTGGGAAAAAAATTCCTCCCGACG------** | -901 |
| -580 | ***SSI*** | **TCCGAATCAGAATCATGTTGCTAAAATTGTTTTAATCCG--------** | -620 |
| -530 | ***SSI*** | **AGATGTCATTATAAACTTTTTTAAAAAAATTCAATAATAT-------** | -490 |
| -518 | ***SSI*** | **TATAATGACATCTATACCTAATAAAAAAATTCGTAAGGTTT------** | -558 |
| -489 | ***SSI*** | **--CTATATTATTGAATTTTTTTAAAAAAGTTTATAATGACATC----** | -529 |
| -438 | ***SSI*** | **-ATTTAACTTTCTACATTTTACAAAAAAAATAAAAAAAAATA-----** | -398 |
| -274 | ***SSI*** | **TAGCTCTCAAATAACTCAATCTAAAATTCGTTCAAATGGA-------** | -314 |
| -228 | ***SSI*** | **CGGATAAATAATACCCTTTTTTAAAAAAGGATTAGTTGTA-------** | -188 |
| -183 | ***SSI*** | **AAACTACAACTAATCCTTTTTTAAAAAAGGGTATTATTT--------** | -223 |
| -181 | ***SSI*** | **TTATTTTTCCATTGTGATTTATAAAAATTGAATTTGTGAA-------** | -141 |
| -153 | ***SSI*** | **-AATTTTTATAAATCACAATGGAAAAATAAATTAAACTACAA-----** | -193 |
| -887 | ***BEI*** | **-CCAAAGAGGACAAGGAAAAACAAAAAGAAGCACAGGAGTAG-----** | -847 |
| -837 | ***BEI*** | **-GCGCGACCAAAGAGGACAAGGAAAAACAAAAAGAAGCACAG-----** | -877 |
| -731 | ***BEI*** | **CCTTTGTGCCGGGAATTAATGGAAAAAGAACCTTAGTTAAG------** | -691 |
| -543 | ***BEI*** | **TAGGCCCTGTTTAGATGGGACTAAAACTTTTAAGTCCCTAT------** | -503 |
| -542 | ***BEI*** | **TAATTATAGATTAATTAGGCTTAAAAAATTTGTCTCGTGAA------** | -502 |
| -541 | ***BEI*** | **-TTTGTATTTATTTGCGTCTGGAAAAAGAAAAGGAAGGAGAG-----** | -501 |
| -457 | ***BEI*** | **--AGTTGAGTACTTAAGAATTCAAAAAAGATTAAGTTCATTGG----** | -497 |
| -269 | ***BEI*** | **CAAAAATCATACGAGGTCCTATAAAATTCGTGTCCTTTTTC------** | -309 |
| -163 | ***BEI*** | **-CTTTGTGCCGGGAATTAATGGAAAAAGAACCTTAGTTAAGC-----** | -123 |
| -112 | ***BEI*** | **-CTCGCACCATTTCCTAGCTACAAAAATACGTTCTCAGTACA-----** | -152 |
| -946 | ***BEIIb*** | **-AATTATAGATTAATTAGGCTTAAAAAATTTGTCTCGTGAAT-----** | -986 |
| -577 | ***BEIIb*** | **--ATTATAGATTAATTAGGCTTAAAAAATTTGTCTCGTGAATT----** | -537 |
| -571 | ***BEIIb*** | **-TTTGTATTTATTTGCGTCTGGAAAAAGAAAAGGAAGGAGAG-----** | -531 |
| -932 | ***ISA1*** | **--TTACCAAAAATTGGTAGGTTAAAAATGTTAATAAAAAAAGC----** | -972 |
| -921 | ***ISA1*** | **--GTTGAAGGTTAAACATTACCAAAAATTGGTAGGTTAAAAAT----** | -961 |
| -848 | ***ISA1*** | **--TGCCAAAATTTAGTAGGGTTAAAAATAACAACAAAGTAAAT----** | -888 |
| -765 | ***ISA1*** | **--GGTAGGTTAAAAATGTTAATAAAAAAAGCAAAGCCCTTAGT----** | -725 |
| -753 | ***ISA1*** | **--TATTAAAATTTGGTAATGCCAAAATTTAGTAGGGTTAAAAA----** | -793 |
| -729 | ***ISA1*** | **--GTTTGGTTTGTTGTCCTATTAAAATTTGGTAATGCCAAAAT----** | -769 |
| -713 | ***ISA1*** | **--GAATGTTCAACATTGCTCACAAAATGTTCTCTTAAATAGTA----** | -673 |
| -697 | ***ISA1*** | **--AGAGCATCTGAATCTGTATTAAAAAAGTACAAAAAAAAACA----** | -657 |
| -681 | ***ISA1*** | **--AATCTGTATTAAAAAAGTACAAAAAAAAACATTCTGAATCT----** | -641 |
| -617 | ***ISA1*** | **--CATTCTGAATCTAGAAAGGGAAAATATCTAGATCTAGAAGC----** | -577 |
| -602 | ***ISA1*** | **--GACTGCACGCGGCCCCCACGAAAAGCCCATGCACGTGGGCC----** | -562 |
| -601 | ***ISA1*** | **--TGGAAGCTTCCTGTTTTTTCAAAATTTGGTAAGATAGCAAT----** | -641 |
| -590 | ***ISA1*** | **--TGCACGTGGGCCCCATCCCGAAAAAAGAGCAACAGCCTCAC----** | -550 |
| -533 | ***ISA1*** | **--GCCCCCCCCCCCCCCAACCCAAAAACCCACGGGCCCCACAC----** | -493 |
| -478 | ***ISA1*** | **--AACTATAAATAATCCACCGGAAAATTCACAATTCGATCGCC----** | -438 |
| -468 | ***ISA1*** | **--GTAATGTTTAACCTTCAACTAAAACAATTTAAACTAAGGAA----** | -428 |
| -439 | ***ISA1*** | **--TGCTATCTTACCAAATTTTGAAAAAACAGGAAGCTTCCACT----** | -399 |
| -398 | ***ISA1*** | **--AACAGGAAGCTTCCACTCTCAAAAACTCTACCAATTTATCC----** | -358 |
| -369 | ***ISA1*** | **--TTTCATCAGTAGATAGCACGAAAACGCCTCATATGACATAA----** | -329 |
| -275 | ***ISA1*** | **--GATATTTATTTGCCTTTAATAAAAATTGAACAAAACACAGC----** | -235 |
| -73 | ***ISA1*** | **--GCCTTTAATAAAAATTGAACAAAACACAGCATTAGCTGTAG----** | -33 |
| -853 | ***SUSIBA2-like*** | **TCTTTACAAATGAGAGACGTCTAAAATTGTTCAATTGCATG------** | -813 |
| -787 | ***SUSIBA2-like*** | **TAGATATAAATCACGCCCCTCTAAAAAATGGAAGCCATATA------** | -747 |
| -726 | ***SUSIBA2-like*** | **ACTAAACTATGGTCGTAATTTTAAAATATGAATAAAATCT-------** | -686 |
| -710 | ***SUSIBA2-like*** | **GACCATAGTTTAGTGTAAGAATAAAAGAAATAGTATAT---------** | -750 |
| -684 | ***SUSIBA2-like*** | **-ATAAGATTTTATTCATATTTTAAAATTACGACCATAGTTTA-----** | -724 |
| -606 | ***SUSIBA2-like*** | **AATTTAACCGCATGTAGAAAGTAAAAAACATTCGCAATATG------** | -566 |
| -942 | ***UGP1*** | **AGCTATGTTTAGTCGCAAGAGGAAAAAATACTAATCTGAC-------** | -982 |
| -462 | ***UGP1*** | **-TTACGACATCTCATGAGAATTAAAAAGTTCGTTAAGGTATT-----** | -502 |
| -365 | ***UGP1*** | **-GACTTTACTTGCTTTTTCTTCAAAAACAACCAGCTTATAAT-----** | -325 |
| -941 | ***SUS4*** | **TGTGTGTCGTACATTATAATGGAAAAAGACATAAATTAG--------** | -981 |
| -813 | ***SUS4*** | **GATGAGTGTGACACAATCCACTAAAAATGATTTGCTTGCC-------** | -773 |
| -726 | ***SUS4*** | **ATATGATATTATTTGATTTTAGAAAAACTTATACGAAGAC-------** | -766 |
| -720 | ***UGP1*** | **AATGAAAATACAGAAACTTCTCAAAAATTAAGCGATCAGTT------** | -680 |
| -687 | ***SUS4*** | **ACGTTATGTTTTACGGGCGGCTAAAAATCTCAGATAACCAA------** | -647 |
| -655 | ***SUS4*** | **TCTGAGATTTTTAGCCGCCCGTAAAACATAACGTGGTAATT------** | -695 |
